# Targeting the circadian negative regulator *period* in *Bombyx mori*: a strategy for improving silk fibroin yield and mechanical properties

**DOI:** 10.1016/j.mtbio.2026.103650

**Published:** 2026-09-04

**Authors:** Guang Wang, Jianfeng Qiu, Jianglan Li, Chenchen Wang, Xinxin Liu, Cheng Luo, Ruji Peng, Taiming Dai, Xiaoning Sun, Yanhong Jiang, Yanghu Sima, Shiqing Xu

**Affiliations:** a School of Life Sciences, Suzhou Medical College, Soochow University, Suzhou, 215123, China; b National Engineering Laboratory for Modern Silk, Soochow University, Suzhou, 215123, China; c College of Bee Science, Fujian Agriculture and Forestry University, Fuzhou, 350002, China

**Keywords:** *Bombyx mori*, Gene editing strategy, Circadian clock, Silk fiber, Production efficiency, Mechanical properties

## Abstract

*Bombyx mori* silk is a natural ultra-long protein fiber with a unique structure and extensive applications. Boosting production efficiency and innovating fiber performance represent critical industry needs as well as major technical bottlenecks. In this study, the mutant of the circadian negative regulator *period* (*per*) showed increased cocoon silk yield and fibroin content by 19.51% and 9.19%, respectively, and reduced silk fineness by 17.53%. Along with increased crystallinity and molecular orientation of fibroin fibers, the tensile strength and toughness of mutant cocoon silk were improved by 50.59% and 46.75%, respectively. Mechanistic analysis revealed that the *per* mutant displayed enhanced posterior silk gland development and fibroin synthesis, thereby increasing cocoon silk yield, particularly fibroin production efficiency. In parallel, the *per* mutant showed enhanced γ-aminobutyric acid (GABA)ergic signaling in the brain of its mature larvae, which was associated with suppressed downstream corazonin secretion and accelerated silk spinning. These findings in the *per* mutant suggest a promising strategy for targeting the circadian system to enhance fibroin production efficiency and mechanical properties of cocoon silk in *B. mori*.

## Highlights

• Identified a potential circadian clock-mediated molecular breeding target acting on the silk gland, a specialized secretory tissue of *B. mori.*
• Silencing *Per* enhances silk production efficiency and fiber mechanical properties, offering a technical example to overcome key bottlenecks.
• The circadian clock regulates silk spinning rate via the GABA-CRZ pathway in silkworms.

## Graphical abstract

**Figure:**
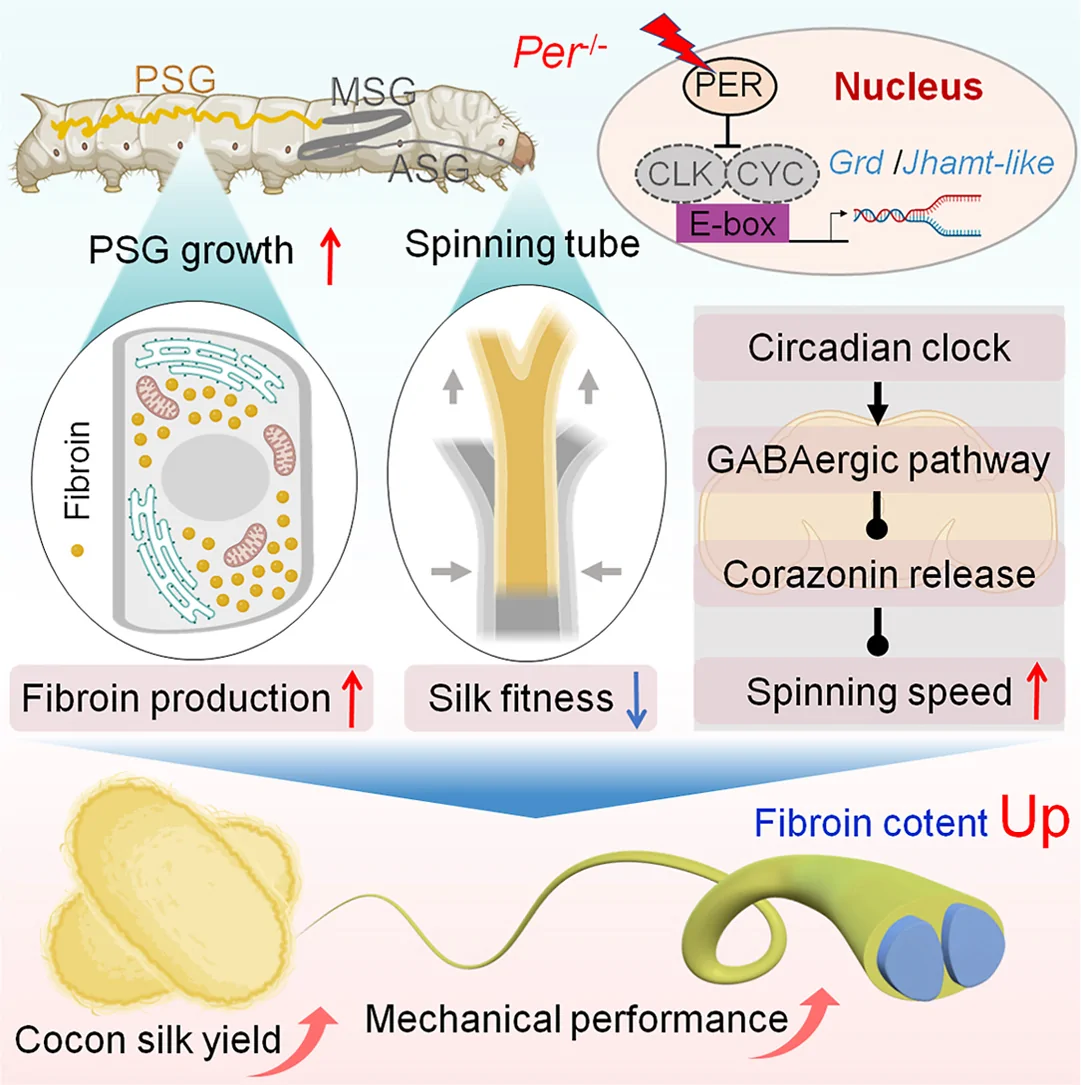

## 1 Introduction

Unlike fibrous materials directly derived from specialized cells, such as wool (animal hair follicles) and cotton (plant seed epidermis), cocoon silk fiber originates from a metastable aqueous protein solution synthesized and secreted into the gland lumen by the silk gland (SG) of *Bombyx mori*. During spinning, tensile force draws the solution into silk fibroin fibers coated with multiple layers of sericin [[1], [2], [3]]. The protein fiber processing unit of the SG is a biological template that textile engineering has yet to surpass, and also endows silk fibers with an unusual combination of toughness, high strength, and high extensibility, as well as the advantages of renewability, excellent biocompatibility, and tunable physicochemical properties [[4], [5], [6], [7]]. As a natural protein fiber, silk's hierarchical molecular architecture underpins its mechanical performance and technological utility [8,9]. These attributes extend the applications of silk fibers beyond traditional high-end textiles to the emerging fields of biomedicine, flexible electronics, and functional materials [[10], [11], [12], [13]]. At present, the demand for silk fibroin in the forms of nonwovens, hydrogels, sponges, and films continues to increase, with consumption already exceeding 35% of total cocoon silk production [[14], [15], [16], [17]]. However, silk production, whether for traditional textiles or emerging applications, utilizes only the fibroin fibers, which account for approximately 70% of the total cocoon silk weight, while the outer sericin proteins are discarded during the reeling process or completely removed by refining [18,19]. Therefore, further research into mechanisms and control technologies to increase the fibroin content of silk is highly anticipated [20,21].

The SG of *B. mori* is a highly specialized organ with the strongest known capacity for protein biosynthesis, synthesizing and secreting silk proteins that account for over 30% of the mature larval body weight [22,23]. Historically, the domestication and breeding of silkworms have focused on balancing improvements to cocoon silk yield and larval robustness, thereby driving the protein synthesis capacity to the very brink of physiological limitations [[24], [25], [26]]. Increasing silk production and especially fibroin fiber production efficiency by further enhancing the protein synthesis capacity of SG cells has thus become a technical bottleneck in sericulture that has been difficult to overcome [6,27,28].

Past studies have clarified numerous important insights into the regulation of SG development, as well as the synthesis and self-assembly of silk proteins [23,[29], [30], [31]]. These advances have facilitated breakthroughs in the targeted editing and expression regulation of genes encoding specific silk protein components across distinct regions of the SG [3,[32], [33], [34], [35]]. Precise regulation of protein synthesis through developmental control or genetic engineering by either enhanced fibroin production in the posterior silk gland (PSG) or reduced sericin synthesis in the middle silk gland (MSG) presents a potential strategy to increase the fibroin content of silk [5,20,21]. However, numerous mutational studies of overexpressed silk protein genes in the PSG have shown that the assembly and secretion of silk fibroin complexes and even the formation of cocoon silk fibers are often adversely affected, leading to abnormal development of the PSG and reduced cocoon silk yield [32,36,37]. Negative effects on cocoon silk yield and silk fiber mechanical properties are also commonly reported in natural flossy cocoon mutants exhibiting degeneration of the MSG, as well as gene-edited silkworm strains with reduced sericin expression in the MSG [[38], [39], [40], [41]]. These findings indicate that strategies to improve fibroin production efficiency via SG gene editing have encountered a significant bottleneck. Comparative analyses of the sericin content of cocoon silk between domesticated and wild silkworms, and among silkworm varieties with different ecological adaptation statuses and those with different cocoon yields, have revealed that a relatively high sericin content benefits the ecological adaptability of silkworms. In contrast, the domesticated silkworm varieties used in current sericulture exhibit a high degree of convergence in the relative contents of silk fibroin and sericin (Tables S1 and S2) [26,[42], [43], [44], [45], [46], [47]]. Therefore, it is necessary to clarify the mechanisms and methods to increase the proportion of silk fibroin fibers while maintaining cocoon yield.

Here, we implemented a strategy that avoids directly introducing exogenous protein genes with severe and unpredictable biological side effects, or overexpressing SG developmental and silk protein-encoding genes. Instead, we used a loss-of-function mutant of the circadian negative regulator *period* (*per*) to gently orchestrate neuroendocrine outputs, thereby reallocating silk protein synthesis resources among different functional regions of the SG and altering the spinning behavior of mature larvae. This approach substantially enhanced the production efficiency and mechanical properties of fibroin fibers in cocoon silk, providing a promising route for the molecular breeding of *B. mori*.

## 2 Materials and methods

### 2.1 Experimental animals

A homozygous mutant (*Per*^−/−^) of the silkworm core circadian gene *per* was previously generated by gene editing [48] and maintained at the School of Life Sciences, Suzhou Medical College, Soochow University. The wild-type (WT) Dazao control strain served as the background genetic line of the mutant. Sanger sequencing and semi-quantitative reverse transcription PCR (RT-PCR) analyses verified that the *Per*^−/−^ mutant contained a 19-base insertion in the target gene, which resulted in a premature stop codon downstream of the target site (Fig. S1), consistent with previous reports. Silkworm larvae were reared on mulberry leaves under controlled conditions (temperature, 25 ± 1.5°C; relative humidity, 75 ± 5%; light:dark cycle, 12:12 h). The cocoon traits, SG development, spinning rate, and mechanical properties of cocoon silk of both male and female larvae were investigated. All other investigations were conducted with only female larvae. In addition, *Per*^−/−^ larvae were injected with 1 nmol corazonin (CRZ) or 10 μg small interfering RNA (siRNA) at approximately 12 h prior to spinning onset, while control *Per*^−/−^ larvae received the same dose of ultrapure water or negative control siRNA (siNC). The CRZ peptide (pQTFQYSRGWTNa) was synthesized by Biodragon Immunology Technology Co., Ltd. (Suzhou, China).

### 2.2 Analysis of cocoon and silk traits and spinning behavior

*Cocoon traits and fibroin/sericin relative contents*: On day 7 after the onset of spinning, the single cocoon weight, cocoon shell weight, and internal pupal weight were measured (*n* = 20 *per* group). After degumming with 0.2% Na_2_CO_3_, dry weights and relative contents of fibroin and sericin in cocoon silk were calculated.

*Cocoon silk fineness*: Individual cocoons were reeled to obtain single fibers (*n* = 20 *per* group). Fiber length and dry weight were determined, and fineness (dtex) was calculated as [cocoon silk dry weight (g)/length (m)] × 10,000.

*Spinning behavior*: The head-swinging frequency of mature larvae during the early spinning stage and silk yield within 24 h were quantified in accordance with a previously described method [49] (*n* = 20 *per* group).

Detailed procedures are provided in the Supplementary Materials and Methods.

### 2.3 Transmission electron microscopy (TEM) and scanning electron microscopy (SEM) observation

The morphologies of cocoon silk within the cocoon layer and degummed silk fibroin fibers were observed by SEM (SU8100, Hitachi, Japan). Samples were sputter-coated with gold for 30 s using an ion sputter coater (MC1000, Hitachi, Japan) at a current of 45 mA. Three independent samples were examined *per* group.

The morphologies of cocoon silk fibers and PSG cells were observed by TEM (HT7800, Hitachi, Japan). The PSG of silkworm larvae on day 3 of the 5th instar stage (L5D3) and silk fibers collected at 24 h after spinning onset were fixed in electron microscopy fixative (R41014, Yuanye Bio-Technology, China) at 4°C for 24 h, followed by post-fixation in 1% osmium tetroxide at room temperature for 2 h. After dehydration in a graded series of ethanol (30%–100%) and 100% acetone, the samples were embedded and cut into ultra-thin sections (Leica EM UC7, Leica Microsystems, Germany), double-stained with 2% uranyl acetate in saturated ethanol solution and 2.6% lead citrate, and then dried prior to observation by TEM.

### 2.4 Determination of the mechanical properties of raw silk thread

The mechanical properties of raw silk thread were tested using a universal tensile testing machine (3365, Instron, USA) in accordance with a previously reported method [5]. Dried cocoons were fully softened and expanded in boiling water. For each group, 20–30 cocoons were used for reeling to produce one continuous raw silk thread at a reeling rate of 75 m/min, maintaining ten cocoons for filament supply throughout reeling. Mechanical testing was performed at 20°C and 65% relative humidity. The initial gauge length was set at 10 mm and a constant tensile rate of 10 mm/min was applied until sample fracture. 30 tensile specimens for mechanical testing were cut from the middle segment of the continuous raw silk thread to exclude unstable initial reeling sections. In addition, raw silk thread diameters were measured under a microscope, with ten readings taken *per* specimen. Average diameters were used to calculate cross-sectional areas (assuming circular cross-section) for normalizing strength and Young's modulus. Tensile properties including toughness were derived from stress-strain curves.

### 2.5 Fourier transform infrared (FTIR) and X-ray diffraction (XRD) analyses

Structural characterization of silk fibroin fiber powder degummed with 0.2% Na_2_CO_3_ was performed in accordance with a previously described method [50,51]. FTIR spectra were collected using a Nicolet™ 380 FT-IR spectrometer (Thermo Fisher Scientific, Waltham, MA, USA) over a range of 4000 to 400 cm^−1^ at a resolution of 4 cm^−1^ with 32 scans. Samples were prepared using the KBr pellet method. Deconvolution and curve fitting of the amide I band (1600–1700 cm^−1^) were performed using PeakFit software (version 4.12; Systat Software GmbH, Erkrath, Germany). XRD patterns were recorded with a SmartLab X-ray diffractometer (Rigaku Corporation, Tokyo, Japan) with Cu Kα radiation, over a 2θ range of 5° to 80° at a scanning rate of 10°/min. The diffractometer was operated at 40 kV and 40 mA. Data were processed using Jade 9 software (Materials Data, Inc., Livermore, CA, USA). After baseline correction and peak fitting, crystallinity (%) was calculated as [total integrated intensity of crystalline peaks/(total integrated intensity of crystalline peaks + area of amorphous halo)] × 100%.

### 2.6 Small-angle X-ray scattering (SAXS) analysis

SAXS measurements of silk samples were performed using a Nano-inXider system (Xenocs SAS, Grenoble, France) equipped with a Cu Kα microfocus source (λ = 1.54 Å), as described previously [6]. Samples were mounted into a sample holder and exposed for 600 s at 25°C with a sample-to-detector distance of 938 mm. Scattering data were collected over a q range of 0–0.45 Å^−1^. The obtained two-dimensional (2D) scattering patterns were processed using FIT2D (European Synchrotron Radiation Facility, Grenoble, France) and OriginPro (OriginLab Corporation, Northampton, MA, USA). The degree of orientation was determined with XSACT software (Xenocs SAS).

### 2.7 EdU (5-ethynyl-2′-deoxyuridine) staining

DNA replication in PSG cells was assessed using the Click-iT™ EdU Cell Proliferation Kit for Imaging, Alexa Fluor™ 594 (C10339, Invitrogen, USA). Silkworm larvae (L5D3) were injected with EdU solution (0.5 mg/mL; 10 μL *per* larva). After incubation for 4 h, PSG tissues were collected, fixed, permeabilized, and stained with Click-iT solution as described previously [32], while the nuclei were counterstained with DAPI (4′,6-diamidino-2-phenylindole). Red (590/615 nm) and blue fluorescence (364/454 nm) were observed under a fluorescence microscope (BX51, Olympus, Japan).

### 2.8 Determination of γ-aminobutyric acid (GABA) content

GABA content was determined with an enzyme-linked immunosorbent assay kit in accordance with the manufacturer's instructions (BA E−2500, LDN, Germany). Brain and hemolymph samples were collected from silkworms at 0, 12, and 24 h after spinning onset. Each group consisted of three biological replicates, with each replicate prepared by pooling whole brains from 15 silkworms or an equal volume of hemolymph from five silkworms. Absorbance at 450 nm was measured using a microplate reader (Eon, BioTek, USA).

### 2.9 Gene expression and functional analysis

*DNA content assay*: PSG and MSG tissues were collected from WT and *Per*^−/−^ silkworm larvae (L5D3), respectively. DNA concentrations were measured by ultraviolet spectrophotometry. Each group consisted of three biological replicates.

*RT-PCR and quantitative real-time PCR (qPCR) analyses*: Total RNA was extracted from SG or brain tissue samples using TRIzol® Reagent (15596018, Thermo Fisher Scientific, USA) and reverse-transcribed into cDNA. Relative mRNA levels were measured by qPCR, while gene transcripts were quantified by RT-PCR after agarose gel electrophoresis. The *Rp49* gene was used as an internal control.

*Transcriptome sequencing and analysis*: PSG tissues were collected from silkworm larvae (L5D3) for transcriptome sequencing. The sequencing data were processed through alignment, quantification, and differential expression analyses to identify differentially expressed genes (DEGs), which were subsequently subjected to Gene Ontology (GO) functional annotation, Kyoto Encyclopedia of Genes and Genomes (KEGG) pathway enrichment analysis, and gene set enrichment analysis (GSEA). Each group consisted of three biological replicates.

*Western blot analysis*: Luminal silk proteins were isolated from intact SGs collected from silkworm larvae at day 6 of the 5th instar (L5D6) and purified. The protein levels of the FIB-H, FIB-L, P25, and the internal reference protein ACTIN of WT and *Per*^−/−^ silkworms were quantified by western blot analysis.

*Gene knockdown*: Female *Per*^−/−^ larvae were collected at 12 h prior to spinning onset. Gene knockdown was performed by microinjection of siRNA targeting the glutamic acid decarboxylase gene (*Gad*), while controls were injected with an equal amount of siNC. Knockdown efficiency was verified by qPCR.

Detailed procedures are provided in the Supplementary Materials and Methods, and the primer sequences are listed in Table S3.

### 2.10 Statistical analysis

Spectral and survival curves were plotted using Origin software. All other statistical analyses and graphing were performed using Prism 8 software (GraphPad Software, LLC, San Diego, CA, USA). Data are presented as the mean ± standard deviation (SD). Comparisons between two groups at each time point were performed with the unpaired Student's *t*-test, followed by the Holm-Sidak correction for multiple comparisons. One-way analysis of variance was used for comparisons among multiple groups. Prior to analysis, percentage data were arcsine square-root transformed to meet normality assumptions. An adjusted probability (*P*) value < 0.05 was considered statistically significant.

## 3 Results

### 3.1 Enhanced cocoon silk yield and silk fibroin production efficiency in the per^−/−^ mutant

**Fig. 1:**
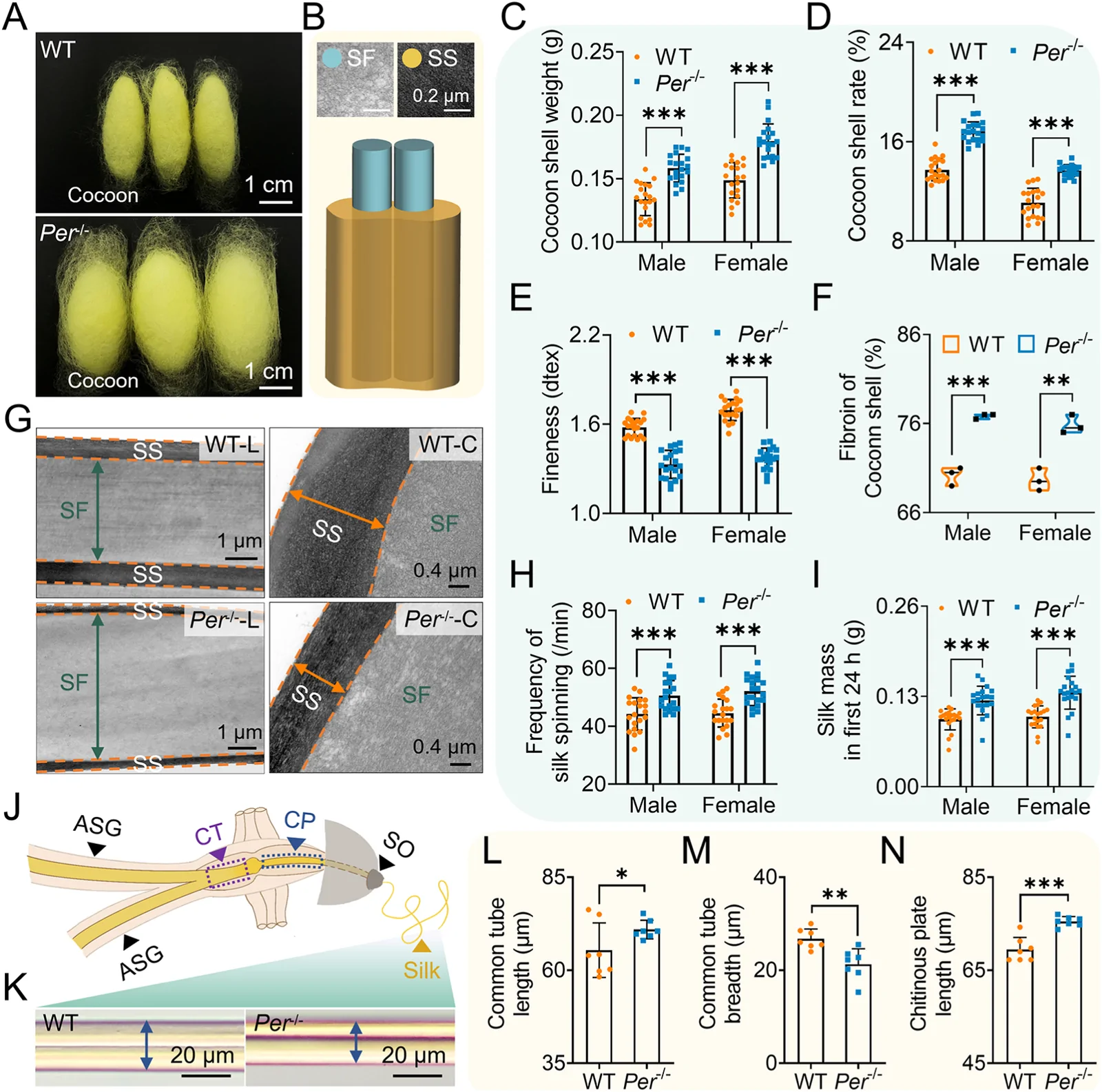
Increased cocoon silk yield and fibroin production efficiency in *Per*^−/−^ silkworms. (A) Cocoon morphology. WT, wild type; *Per*^−/−^, *period* knockout mutant. (B) Schematic diagram of cocoon silk structure. The silk core consists of long hydrophobic silk fibroin fibrils (SF), whereas the outer layer is composed of water-soluble silk sericin (SS). (C) Cocoon shell weight. (D) Cocoon shell rate. (E) Cocoon silk fineness. For C–E, *n* = 20. (F) Relative fibroin content of cocoon silk. *n* = 3. (G) TEM images of cross and longitudinal sections of cocoon silk. L, longitudinal section; C, cross section. (H) Head-swinging frequency of silkworm larvae during the silk spinning stage. *n* = 20. (I) Silk mass secreted within 24 h of spinning onset. *n* = 20. (J) Schematic diagram of the silkworm spinneret. SO, spinneret orifice; CP, chitinous plate; CT, common tube; ASG, anterior silk gland. (K) Optical micrograph of cocoon silk. (L) Common tube length. (M) Common tube breadth. (N) Chitinous plate length. For L–N, *n* = 7. The spinneret was dissected 6 h after spinning onset. Data are presented as mean ± SD. *P ≤ 0.05, **P ≤ 0.01, ***P ≤ 0.001.

Following purification and identification over multiple generations, the circadian gene *period* knockout mutant (*Per*^−/−^) silkworms exhibited stable economic traits comparable to the WT, including larval viability, growth, and cocooning performance (Fig. S2A–E). Surprisingly, the mutant produced significantly larger cocoons (Fig. 1A), as the cocoon shell weight of males and females was increased by 18.30% and 20.71%, respectively, as compared to the WT (Fig. 1C), while the cocoon shell rate, an indicator of silk production efficiency, was increased by 22.25% and 23.35% (Fig. 1D), respectively, whereas the cocoon silk fineness, a measure of silk quality, was 15.79% and 19.26%, respectively, which was finer than that of the WT (Fig. 1E). Notably, the relative content of silk fibroin, the effectively utilized silk protein component in silk production, was 9.51% and 8.86% higher in males and females, respectively, than in the WT (Fig. 1F), while the relative content of sericin, a waste product, was correspondingly reduced (Fig. S2H). TEM observation of the silk fiber ultrastructure further confirmed this important change (Fig. 1G and S3A). Statistical analysis showed that the mutant had a significantly greater proportion of silk fibroin layer area as compared to the WT (Fig. S3B and C). Together, these results showed that the *Per*^−/−^ mutant exhibited markedly elevated cocoon silk yield and silk fibroin protein production efficiency.

Further investigation into the spinning behavior closely related to silk fibroin/sericin assembly revealed that the mature *Per*^−/−^ larvae exhibited a higher head-swinging frequency during the spinning stage (Fig. 1H and Video S1). The silk mass secreted in the first 24 h of spinning exceeded that of the WT by 31.77% (Fig. 1I), accounting for 88.91% of the total silk secretion, while the WT reached only 78.55% during the same spinning period (Fig. S2I and J). Analysis of spinneret morphology (Fig. 1J and S3D), which is closely related to the silk fibrosis process, revealed that the length of the common tube, which regulates silk fluid flow rate and cocoon silk fineness, was 8.47% greater for the mature *Per*^−/−^ larvae than the WT, while the width had decreased by 20.43% (Fig. 1L and M). Additionally, the length of the chitinous plate in the pressing zone was significantly increased by 8.69% (Fig. 1N).

Supplementary data related to this article can be found online at https://doi.org/10.1016/j.mtbio.2026.103650

The following are the Supplementary data related to this article:

Multimedia component 1

**Fig. 2:**
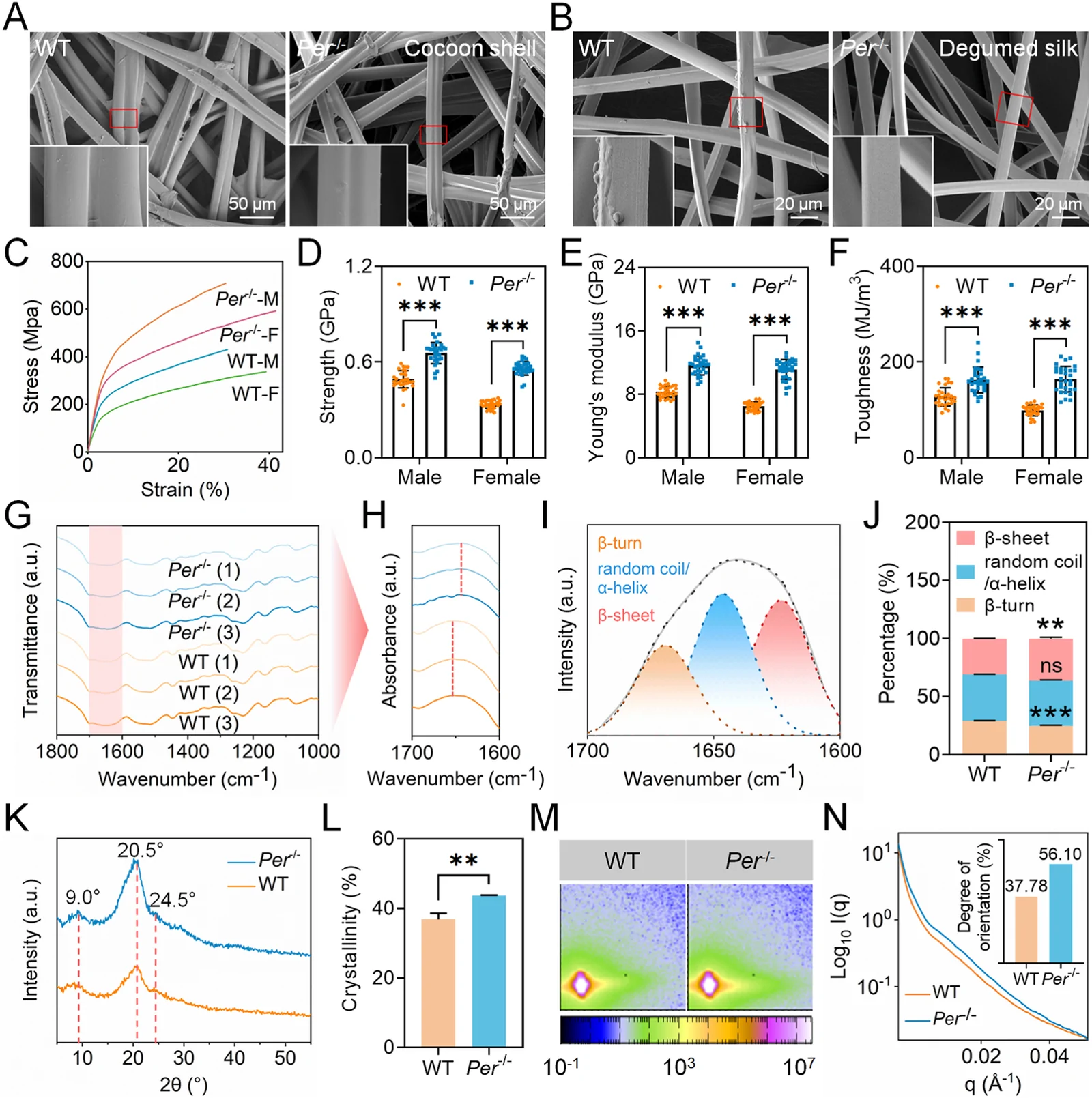
Enhanced mechanical properties of raw silk threads from *Per*^−/−^ silkworms. SEM images of (A) cocoon silk and (B) degummed fibroin fibers. The samples were prepared from the middle layer of the cocoon and from silk fibers degummed by boiling in 0.2% Na_2_CO_3_ for 30 min. (C–F) Mechanical properties of raw silk threads. M, Male; F, Female. *n* = 30. (C) Stress-strain curves. (D) Tensile strength. (E) Young's modulus. (F) Toughness. (G) FTIR spectra of silk fibers. The red shaded region (1700–1600 cm^−1^) represents the amide I band. (H) Magnified view of the amide I band. The absorption peak of the amide I band was shifted in the *Per*^−/−^ mutant as compared to the WT. (I) Example deconvolution of the amide I band. Yellow, blue, and red absorption peaks represent β-turn, random coil/α-helix, and β-sheet, respectively. (J) Relative contents of secondary structures of silk fibroin. (K) XRD patterns of silk fibers. (L) Crystallinity. The characteristic diffraction peaks at 9°, 20.5°, and 24.5° were used for deconvolution and fitting to determine crystallinity. Three independent experimental replicates were included for both FTIR and XRD analyses. (M) The 2D SAXS patterns of silk fibers. The equatorial scattering of the *Per*^−/−^ sample is significantly enhanced, indicating higher orientational order of nanofibers. (N) Comparison of SAXS intensity profiles of silk fibers in the q range of 0.008–0.06 Å^−1^. Data are presented as mean ± SD. ns, not significant (*P* > 0.05); *P ≤ 0.05, **P ≤ 0.01, ***P ≤ 0.001. (For interpretation of the references to colour in this figure legend, the reader is referred to the Web version of this article.)

The structure and mechanical properties of silk fibers from the *Per*^−/−^ silkworms were analyzed. SEM characterization revealed that the *Per*^−/−^ group exhibited larger pores and reduced adhesion between cocoon filaments, along with finer cocoon filaments (Fig. 2A), consistent with the fineness measurements. Additionally, under the same degumming conditions, the surface of the cocoon filaments was smoother in the *Per*^−/−^ group (Fig. 2B). Testing of the mechanical properties showed that, as compared to the WT group, raw silk threads produced by males and females in the *Per*^−/−^ group exhibited significantly greater strength (33.27% and 67.91%, respectively), Young's modulus (40.05% and 70.90%, respectively), and toughness (27.63% and 65.86%, respectively), while there was no significant difference in elongation at breakage (Fig. 2C–F and S3E). These results showed that *Per*^−/−^ silkworms exhibited improved silk mechanical properties, with a stronger effect in females, and these changes were associated with alterations to spinneret morphology and spinning rate.

FTIR spectroscopy showed that silk fibroin fibers of the WT and *Per*^−/−^ groups exhibited fundamentally similar absorption peak positions and numbers, but shifts in peak position and differences in relative intensity were observed in the amide I region (1700–1600 cm^−1^), indicating differences in the secondary structure of silk fibroin between the two groups (Fig. 2G and H). Deconvolution analysis of this band further confirmed that the β-sheet content was significantly increased in the *Per*^−/−^ group as compared to the WT group (Fig. 2I and J). The XRD results showed that both groups exhibited the typical crystalline diffraction peaks of silk fibroin at approximately 9.0°, 20.4°, and 24.5°. Peak fitting analysis revealed that the *Per*^−/−^ group had a crystallinity of 43.64%, significantly higher than the WT group of 36.87% (Fig. 2K and L). Furthermore, 2D SAXS analysis revealed that both groups of silk fibers exhibited anisotropic scattering characteristics, while there were differences in electron density distributions (Fig. 2M). The one-dimensional scattering curves obtained by radial integration of the 2D images revealed distinct differences in scattering intensity distribution within a q range of 0.008–0.06 Å^−1^ (Fig. S3F). Orientation analysis further indicated a higher structural order of cocoon silk in the *Per*^−/−^ group than the WT group, with the degree of orientation increasing from 37.78% in the WT group to 56.10% in the *Per*^−/−^ group (Fig. 2N). These results indicated that silk fibers from the *Per*^−/−^ silkworms displayed elevated β-sheet nanocrystals content and orientation, which correlated with increased strength and toughness.

### 3.2 Enhanced PSG development and fibroin synthesis and secretion in the per^−/−^ mutant

**Fig. 3:**
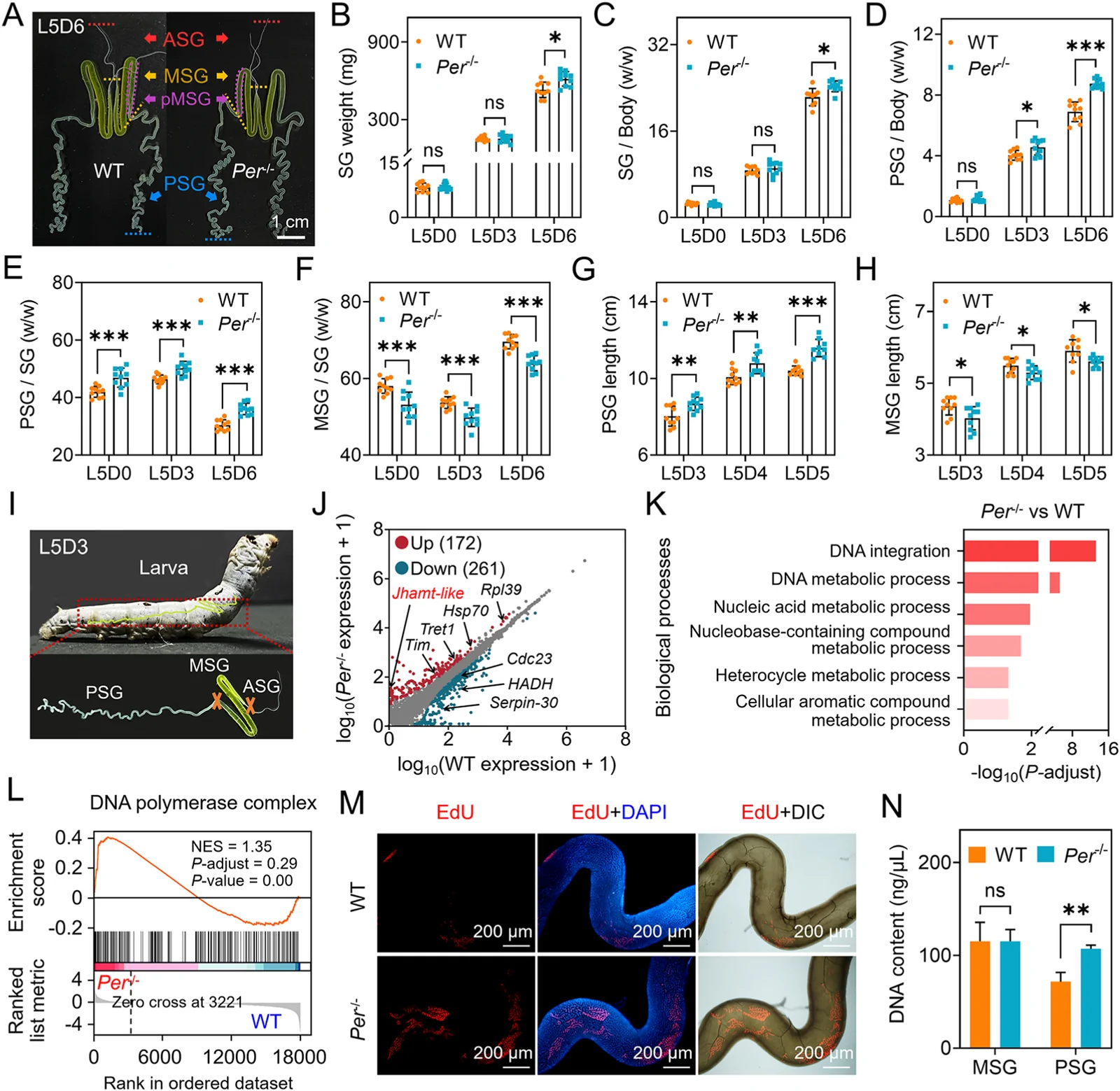
Enhanced development and endoreplication of the PSG in *Per*^−/−^ silkworms. (A) Morphology of SGs. ASG, anterior SG; MSG, middle SG; pMSG, posterior MSG; PSG, posterior SG. (B) SG weight. (C) SG-to-body weight ratio. (D) PSG-to-body weight ratio. (E) PSG-to-SG weight ratio. (F) MSG-to-SG weight ratio. (G) PSG length. (H) MSG length. For B–H, *n* = 10. (I) PSG (L5D3). (J) DEGs of the PSG between the *Per*^−/−^ and WT groups. Red and blue represent significantly upregulated and downregulated genes of the *Per*^−/−^ group as compared to the WT group, respectively (adjusted *P* < 0.05). *n* = 3. (K) GO analysis of DEGs. (L) GSEA. (M) EdU-DAPI double staining of the PSG at L5D3 with labeling replicating nuclei (EdU) and total nuclei (DAPI), respectively. (N) DNA contents of the MSG and PSG at L5D3. *n* = 3. Note: L5D0, L5D3, L5D4, L5D5, and L5D6 represent days 0, 3, 4, 5, and 6 of the 5th instar stage, respectively. Data are presented as mean ± SD. ns, *P* > 0.05; *P ≤ 0.05, **P ≤ 0.01, ***P ≤ 0.001. (For interpretation of the references to colour in this figure legend, the reader is referred to the Web version of this article.)

We investigated the mechanism basis for elevated cocoon silk production efficiency in the *Per*^−/−^ silkworms, focusing on SG developmental changes in the 5th instar stage, characterized by rapid endoreduplication of SG cells and active silk protein synthesis (Fig. 3A). Morphological observation showed that the SG of both *Per*^−/−^ and WT silkworms were fully developed and structurally intact (Fig. S4A). The SG weight and SG/body weight ratio were similar between the *Per*^−/−^ and WT groups at day 0 (L5D0) and day 3 (L5D3) of the 5th instar, but were significantly higher in the *Per*^−/−^ group at L5D6 (Fig. 3B and C), which might have been attributed to significantly increased PSG/body weight ratio during the middle and late stages (Fig. 3D). Notably, the relative weight ratio of the PSG, which specifically synthesizes silk fibroin, to the whole SG was significantly higher in the *Per*^−/−^ group than the WT group throughout this investigation, whereas that of the MSG, which specifically synthesizes silk sericin, was significantly lower (Fig. 3E and F). Both sexes showed consistent trends, indicating a genetic trait (Fig. S4B–F). Changes to SG length were further examined during the middle and late 5th instar stages, when silk protein synthesis is most active. Consistently, the PSG length was greater in the *Per*^−/−^ group than the WT group, whereas the MSG length was significantly shorter, with the difference mainly occurring in the posterior region of the MSG (Fig. 3G, H and S4G). These results showed that *Per*^−/−^ silkworms exhibited remodeled developmental pattern of the SG, with opposing phenotypic alterations between the PSG and MSG.

Transcriptomic sequencing was performed to analyze gene expression changes in the PSG of L5D3, when silk protein synthesis begins to accelerate, and revealed that the *Per*^−/−^ mutant had 172 upregulated and 261 downregulated genes as compared to the WT (Fig. 3I and J). These genes were mainly enriched in pathways associated with DNA replication (Fig. 3K, L and S5A–C). EdU staining showed more active DNA replication in the PSG of *Per*^−/−^ silkworms (Fig. 3M and S5D). Consistently, the DNA content of the PSG was significantly higher for the *Per*^−/−^ mutant than the WT at L5D3, whereas there was no significant difference in the DNA content of the MSG between the two groups (Fig. 3N). These results suggested that enhanced PSG development and fibroin synthesis were observed in the *Per*^−/−^ mutant.

**Fig. 4:**
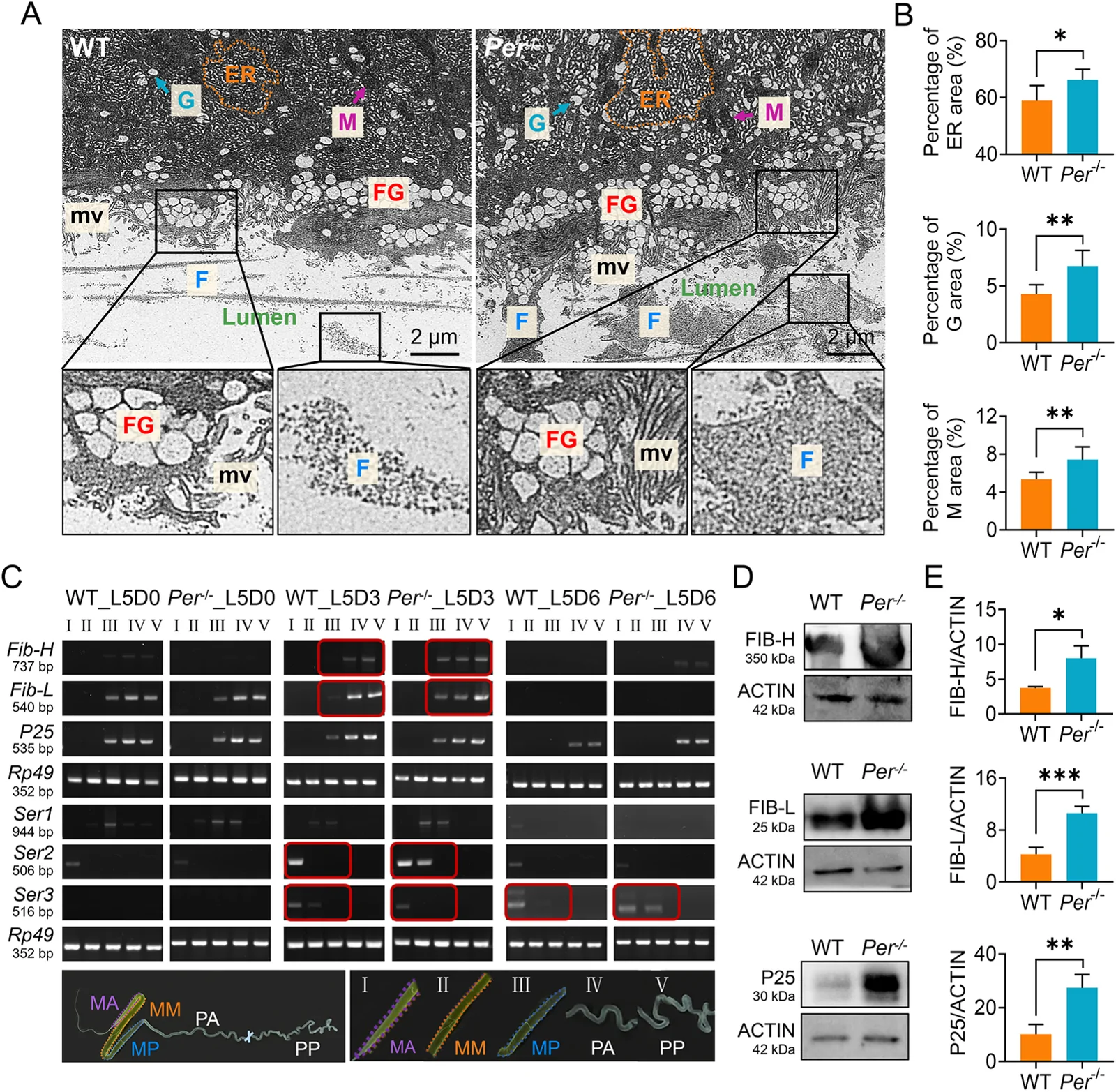
Increased synthesis and secretion of fibroin in *Per*^−/−^ silkworms. (A) TEM images of PSG cells. Samples were collected from the middle region of the PSG (L5D3). G, Golgi apparatus; ER, endoplasmic reticulum; M, mitochondrion; FG, fibroin globules; mv, microvilli; F, fibroin in the lumen. (B) Quantitative analysis of the relative areas of subcellular organelles involved in silk protein synthesis in PSG cells. The areas of the ER, M, and G in TEM images were quantified using ImageJ software. (C) RT-PCR was used to examine transcript levels of silk protein genes in different SG regions. MA, MM, and MP represent the anterior, middle, and posterior regions of the MSG, respectively. PA and PP represent the anterior and posterior regions of the PSG, respectively. *Rp49* served as the internal reference gene. (D, E) Fibroin protein levels in the SG lumen. Western blot analysis of FIB-H, FIB-L, and P25 in the SG lumen of L5D6. ACTIN served as the internal reference. *n* = 3. Data are presented as mean ± SD. *P ≤ 0.05, **P ≤ 0.01, ***P ≤ 0.001.

TEM was used to examine the ultrastructure and fibroin secretion of PSG cells of L5D3. In *Per*^−/−^ silkworms, subcellular organelles involved in silk protein synthesis, including the rough endoplasmic reticulum, Golgi apparatus, and mitochondria, were significantly enriched in PSG cells, and more fibroin molecules were secreted into the PSG lumen as compared to the WT (Fig. 4A and B).

RT-PCR was used to analyze the spatiotemporal expression of silk protein genes in different SG regions. The results showed that the expression patterns of fibroin heavy chain (*Fib-H*), fibroin light chain (*Fib-L*), and *P25* were consistent between the *Per*^−/−^ and WT groups at L5D0 and L5D6. However, at L5D3, *Fib-H* and *Fib-L* were expressed in the anterior and posterior regions of the PSG, as in the WT, but were also ectopically expressed in the posterior region of the MSG, where no expression was detected in the WT. Additionally, *Ser1* expression levels were higher in the anterior region of the PSG of the *Per*^−/−^ mutants than the WT at L5D0, as well as in the middle and posterior regions of the MSG at L5D3. Furthermore, *Ser2* and *Ser3*, which were undetectable in the middle MSG region of the WT, were highly expressed in the *Per*^−/−^ mutants at L5D3 and L5D6, respectively (Fig. 4C). These results indicated that the strict spatiotemporal expression profiles of major silk protein genes were altered in the mutant. The reduced sericin content in cocoon silk was not caused by downregulation or silencing of the sericin gene in functional SG regions, but rather by the combined effects of a reduced proportion of sericin gene transcripts in the entire SG and upregulated fibroin gene transcription.

KEGG enrichment analysis revealed that the only significantly enriched pathway among the upregulated genes in the PSG of the *Per*^−/−^ mutants at L5D3 was "Protein processing in endoplasmic reticulum" (Fig. S5E). Consistently, the transcription levels of *Ugt* and *Manl*, which are involved in protein processing in the endoplasmic reticulum, were significantly upregulated in the SG cells of *Per*^−/−^ mutants during the mid-to-late 5th instar stage (Fig. S6C and D). Furthermore, western blot analysis was used to detect the levels of fibroin proteins synthesized by SG cells and secreted into the lumen at L5D6. The results revealed that the protein levels of FIB-H, FIB-L, and P25 were significantly higher in the *Per*^−/−^ group than the WT group (Fig. 4D). Furthermore, at 0 h after the initiation of spinning, the PSG/body weight ratio was significantly higher in the *Per*^−/−^ group than the WT group, while no significant difference was observed in the MSG/body weight ratio at this time point. However, both ratios were significantly lower in the *Per*^−/−^ group than the WT group at 24 h after spinning onset (Fig. S6E–H), supporting the higher relative fibroin content and faster spinning rate of *Per*^−/−^ mutants (Fig. 1F–I). These results showed that enhanced fibroin synthesis and secretion in the SG of *Per*^−/−^ mutants.

### *3.3* *Accelerated spinning rate associated with the GABA-CRZ pathway in the per*^−/−^*mutant*

**Fig. 5:**
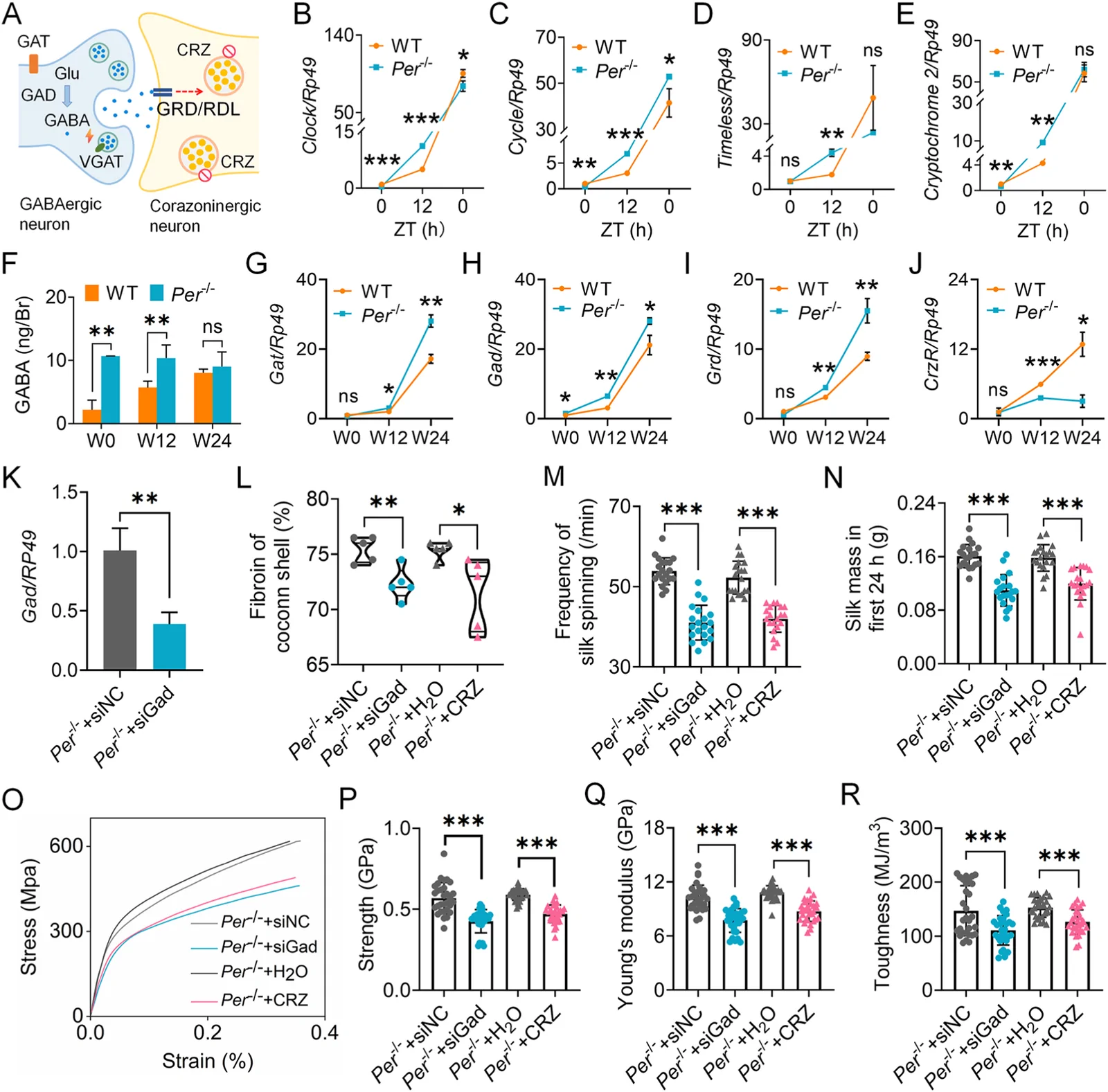
GABAergic signaling modulates the spinning rate of mature *Bombyx mori* larvae. (A) Schematic diagram of GABAergic signaling. Glu, L-glutamate; GAT, GABA transporter; GAD, glutamate decarboxylase; GRD, ionotropic GABA receptor; RDL, GABA-gated chloride channel; VGAT, vesicular GABA transporter. (B–E) Transcript levels of core circadian TTFL genes in the silkworm brain during the spinning stage. ZT0 was set as 0 h after spinning onset, and samples were collected every 12 h. Transcript levels in the brain at 0, 12, and 24 h after spinning onset were determined by qPCR. *Rp49* served as the internal reference gene. *n* = 3. (F) GABA content in the silkworm brain during the spinning stage. (G–J) Transcript levels of the GABA synthesis rate-limiting enzyme genes *Gad* and *Gat*, GABA receptor gene *Grd*, and CRZ receptor gene *CrzR*. *n* = 3. (K, L) Effects of *Gad* knockdown or CRZ supplementation on fibroin content in cocoon silk. *Per*^−/−^ female larvae of similar body size were randomly selected and injected with 10 μg siGad or 1 nmol CRZ at 12 h before spinning onset (after feeding cessation). Control larvae received an equal volume of siNC or ultrapure water. (K) Transcript levels of *Gad* in the brain at 24 h after siRNA injection determined by qPCR. *n* = 3. (L) Relative fibroin content in cocoon silk. *n* = 5. (M, N) Effects of *Gad* knockdown or CRZ supplementation on the spinning rate. (M) Head-swinging frequency during the spinning stage. (N) Silk mass within 24 h of spinning onset. (O–R) Effects of *Gad* knockdown or CRZ supplementation on the mechanical properties of raw silk threads. *n* = 30. (O) Stress-strain curves. (P) Tensile strength. (Q) Young's modulus. (R) Toughness. Data are presented as mean ± SD. ns, *P* > 0.05; *P ≤ 0.05, **P ≤ 0.01, ***P ≤ 0.001.

Our previous study found that the circadian transcription factors CLOCK and CYCLE positively regulate transcription of the GABA receptor gene *Grd* [48], and that GABA inhibits release of the neuropeptide CRZ, which negatively regulates the spinning rate of silkworms [49,52] (Fig. 5A). To investigate the mechanism underlying the increased spinning rate of the mutant and the potential impact on silk fiber mechanical properties, changes to the expression levels of core clock genes within the circadian transcription-translation feedback loops (TTFLs) during the first 24 h after the initiation of spinning were examined. The results showed that at 12 h after spinning onset, the transcript levels of the major TTFL components *Clock*, *Cycle*, *Timeless*, and *Cryptochrome 2* were significantly upregulated in the brain tissue of the mutant group as compared to the WT group (Fig. 5B–E), suggesting that the circadian system may have influenced the spinning rate through neuropeptide signaling.

Next, the GABA content of the silkworm brain during the spinning stage was assessed to determine whether the increased spinning rate of the *Per*^−/−^ mutant was related to GABA-regulated release of CRZ. The results showed that the brain GABA content of the WT group gradually increased from 0 to 24 h after spinning onset, whereas the content of the *Per*^−/−^ brain was consistently higher (Fig. 5F). Examination of transcript levels of GABA synthesis-related and receptor genes showed that at 0 h after spinning onset, *Gad* and *Rdl1* were significantly upregulated, while *Vgat* was downregulated as compared to the WT group, with no significant difference in the transcript levels of *Grd* and *Rdl2*. In contrast, at 12 and 24 h after spinning onset, each of these genes were significantly upregulated relative to the WT group, whereas the CRZ receptor gene *CrzR*, downstream of GABAergic neurons, was significantly downregulated (Fig. 5G–J and S7B–D). These results suggested that enhanced GABAergic signaling in the mutant silkworm brain influenced the downstream action of CRZ.

We next focused on the role of GABA-CRZ signaling in the altered spinning rate and cocoon silk properties of *Per*^−/−^ silkworms. As compared to the negative control (siNC), siGad significantly reduced *Gad* transcript levels in the brains of *Per*^−/−^ silkworms during the spinning stage (Fig. 5K). Analysis of silk fiber composition showed that knockdown of *Gad* or supplementation of CRZ decreased the relative fibroin content in *Per*^−/−^ cocoons by 3.20% and 3.90%, respectively, while the sericin content was increased (Fig. 5L and S7E, F). Analysis of the spinning rate revealed that both *Gad* knockdown and CRZ supplementation significantly reduced the spinning rate of *Per*^−/−^ silkworms (Fig. 5M, Videos S2 and S3) without altering the total silk production (Fig. S7G). The mass and proportion of silk secreted during the first 24 h of spinning were significantly decreased (Fig. 5N and S7H), possibly because the reduced spinning rate prolonged the attachment and curing time of water-soluble sericin, leading to increased sericin deposition on the outer layer of silk fibers. More importantly, knockdown of *Gad* or supplementation of CRZ resulted in significant decreases in the tensile strength, Young's modulus, and toughness of the raw silk threads (Fig. 5O–R). These results support a working model in which altered GABA-CRZ signaling in the *Per*^−/−^ mutant is associated with the accelerated spinning rate and may contribute to the improved mechanical properties of raw silk threads.

Supplementary data related to this article can be found online at https://doi.org/10.1016/j.mtbio.2026.103650

The following are the Supplementary data related to this article:

Video 2

Video 3

## 4 Discussion

### 4.1 Improving cocoon silk production efficiency through the circadian system is a unique and effective approach

The circadian clock of *B. mori* plays a key regulatory role in rhythmic phenotypes, such as egg hatching, diapause, and eclosion, and also affects basic phenotypes like the growth rate. However, the loss of different TTFL components results in distinct phenotypic outcomes with significantly differing effects [[53], [54], [55]]. The mechanisms involve the TTFL regulatory pathway, and also noncanonical functions exerted by clock proteins, either independently or in coordination with other signaling pathways. Specifically, these proteins modulate diapause determination, developmental rate, energy metabolism, and stress resistance through hormone effects involving diapause hormone, molting hormone, and juvenile hormone (JH) [48,56,57], illustrating the diverse regulatory modes underlying these phenotypes.

In lepidopteran insects, such as *B. mori*, the core circadian oscillator operates through TTFLs composed of multiple functional components, including the positive regulators CLOCK and CYCLE, the negative regulators PER and CRYPTOCHROME 2, and the photoreceptor CRYPTOCHROME 1. These TTFLs regulate the expression of clock genes and downstream clock-controlled genes, thereby widely affecting rhythm-related physiological processes [56,58]. Early studies generally suggested that larval feeding, development of specialized SGs, and silk protein synthesis in silkworms were weakly influenced by circadian rhythms [[59], [60], [61]]. However, a recent study showed that silkworms carrying mutations in the *Clock* gene, a positive regulator of TTFLs, exhibited prolonged developmental duration, 25% higher pupal weight, and 7% greater cocoon silk yield, whereas the cocoon shell rate was reduced [62]. In this study, mutant silkworms lacking functional *per* expression, a negative regulator of the TTFLs, displayed significant increases in cocoon silk yield, cocoon shell rate, and silk fibroin production efficiency, without accompanying abnormalities in the viability or developmental rate. This is a promising finding, suggesting that *per*, a core component of the circadian clock, represents a potential molecular target to precisely manipulate the cocoon silk yield and efficiency-related phenotypes. However, further investigations are needed to determine whether this effect arises from the regulatory role of the circadian TTFLs or the pleiotropic effects of clock genes via noncanonical functions. All phenotypic data in this work were obtained from the laboratory strain Dazao. We expect the beneficial effects of loss of *per* function to be generally reproducible in commercial strains, although the magnitude of improvement in cocoon traits may vary with genetic background. Future work should test this in commercial strains such as 932, 7532, and their hybrids.

### 4.2 Mechanisms underlying enhanced silk fibroin production efficiency following loss of *per* function

Although SG cells do not increase in number after the embryonic stage, the PSG and MSG undergo approximately 20 rounds of endoreduplication during the larval stage, resulting in astronomical amplification of silk protein gene copy numbers in the nucleus [29]. Such amplification forms the cytological basis for the high-level transcription of silk protein genes in SG cells, which ultimately determines the cocoon silk yield [23,28]. SG development and endoreduplication are regulated by molting hormone and JH. Exogenous supplementation of these hormones at different time points during the 5th instar stage produces distinct effects, reflecting their differential roles under varying physiological states and complex hormonal interplay [5,63,64]. Our previous studies have demonstrated that the circadian transcription factors, CYCLE/CLOCK, regulate JH biosynthesis by modulating expression of the *Jhamt-like* gene [57]. JH has been shown to positively regulate SG development and silk protein expression [65,66]. In addition, the SG factors, Dimm, Sage, and Sgf2, are also involved in regulating fibroin gene transcription [31,67,68]. In this study, loss of *per* function promoted PSG development and increased the DNA reserve for fibroin synthesis, while the transcript levels of *Dimm*, *Sage*, and *Sgf2* were significantly upregulated (Fig. S5H). Meanwhile, subcellular organelles related to protein synthesis were significantly enriched in PSG cells, and fibroin secretion was increased in the SG lumen. Transcriptome analysis of PSG cells from *Per*^−/−^ silkworms revealed that *Jhamt-like* mRNA was significantly upregulated (Fig. S6A). Furthermore, the transcript levels of the JH receptor *Met2* and its downstream target gene *Kr-h1* were also elevated in the SG cells of L5D3 *Per*^−/−^ mutants (Fig. S6B). On the basis of these transcriptional signatures alone, we hypothesize that JH signaling could contribute to elevated fibroin synthesis and secretion in *Per*^−/−^ silkworms, although this awaits further functional validation. Notably, MSG development and sericin synthesis of *Per*^−/−^ silkworms did not exhibit the same promoting effects as observed in the PSG, suggesting differences in the endocrine signaling pathways governing endoreduplication between the PSG and MSG. In addition, ectopic expression of *Fib-H* and *Fib-L* in the posterior MSG of *Per*^−/−^ silkworms reflects disruption of the strict spatiotemporal patterning of core silk protein genes. Whole SG transcript analysis revealed elevated fibroin transcripts and reduced sericin transcripts in *Per*^−/−^ silkworms (Fig. S5F and G). Enhanced PSG development and increased fibroin synthesis within the PSG, along with this ectopic fibroin expression, contribute to the marked increase in fibroin proportion.

**Fig. 6:**
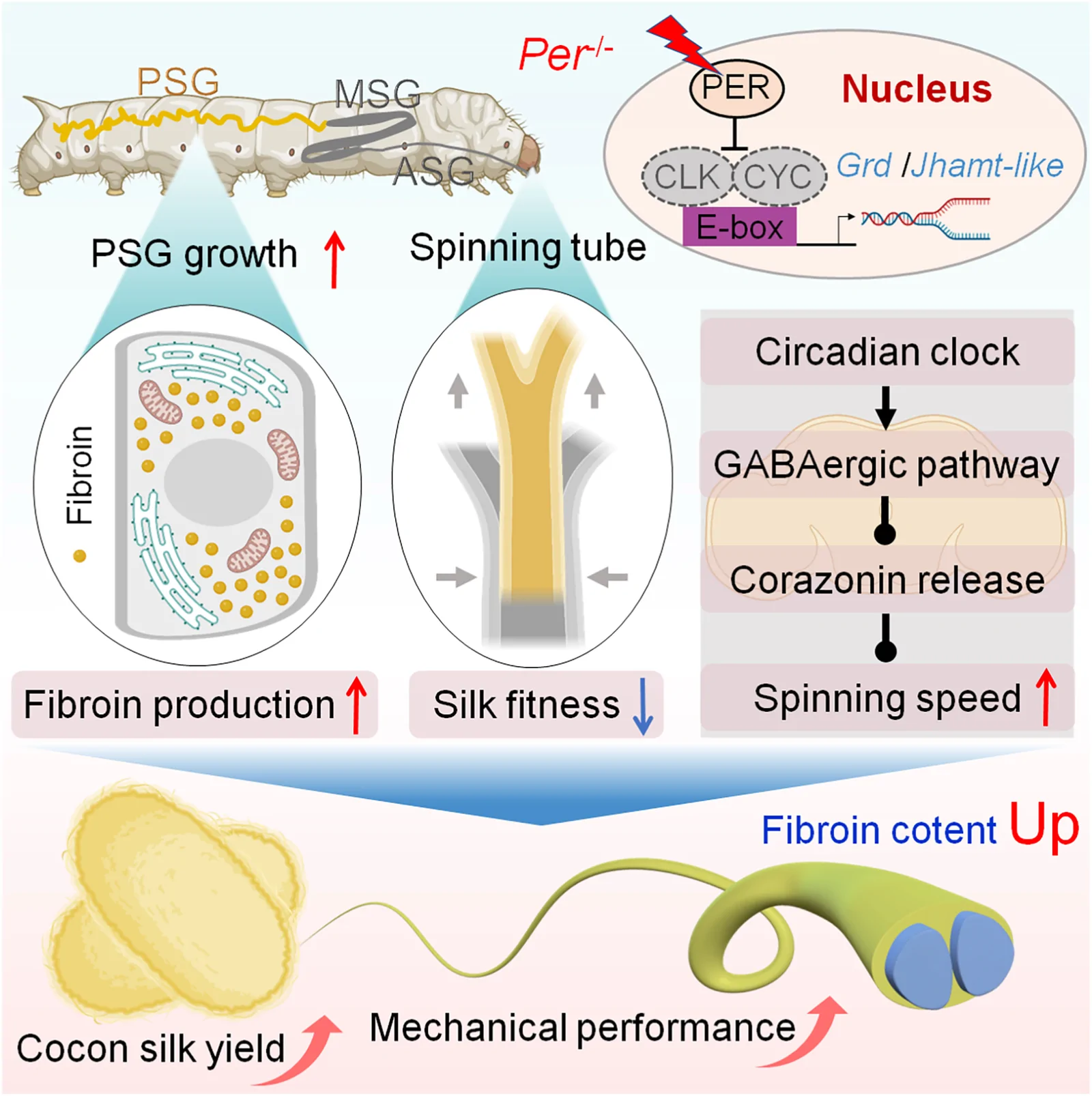
Schematic model of enhanced fibroin production efficiency and mechanical properties of cocoon silk observed in the *Per*^−/−^ mutant. Loss-of-function mutation of *per*, a negative regulator of the circadian transcriptional activators CLK (CLOCK) and CYC (CYCLE), upregulated transcription of the downstream target genes *Grd* (GABA receptor) and *Jhamt-like*. In *Per*^−/−^ mutant silkworms, fibroin synthesis in the PSG was specifically enhanced, accompanied by a narrower and longer spinning tube, resulting in cocoon silk of finer denier. During the spinning stage, enhanced brain GABAergic signaling in *Per*^−/−^ mutants is putatively linked to reduced corazonin release, a negative regulator of the spinning rate, and to the accelerated spinning rate. These synergistic processes ultimately increased the fibroin proportion in mutant cocoon silk from 69.92% in the WT to 76.33%, while simultaneously improving the cocoon silk yield and mechanical properties. Red arrows indicate increase, blue arrows indicate decrease, and black arrows and water droplets represent promoting and inhibitory signals, respectively. (For interpretation of the references to colour in this figure legend, the reader is referred to the Web version of this article.)

Previous studies have demonstrated that during the silk spinning period, the ultra-high concentrations of metastable liquid silk proteins in the SG lumen are extruded through the spinning tube comprising the ASG and spinneret, and rapidly solidify into silk fibers under the combined action of shear stress and mechanical traction [23,69,70]. The spinneret structure and spinning behavior of the silkworm play a decisive role in determining the fineness and strength of silk fibers [2,71,72]. Numerous studies have shown that forcibly increasing the spinning rate or adding graphene, carbon nanotubes, and calcium lignosulfonate to silkworm feed significantly improve the mechanical properties of silk fibers. However, these methods still face efficiency and cost challenges in large-scale silk production [50,[73], [74], [75]]. In this study (Fig. 6), loss of *per* function led to a narrower and longer spinning tube. Consequently, the silk protein solution was subjected to a higher shear rate when passed from the common duct into the pressing zone. Meanwhile, the higher head-swinging frequency during the spinning stage further reduced the cocoon filament fineness by accelerating the spinning rate, particularly decreasing the thickness of the water-soluble sericin layer adhering to the fibroin fiber surface. Consequently, the narrower and longer spinning tube, combined with increased extrusion pressure in the pressing zone and accelerated spinning rate, resulted in a thinner sericin layer on the outer surface of the rapidly solidified fibroin fibers, leading to an increased fibroin protein content of the cocoon filaments. Furthermore, our data suggest that the accelerated spinning rate observed in *Per*^−/−^ silkworms may be associated with altered GABA-CRZ signaling. The resulting strong shear force improved the β-sheet content, crystallinity, and molecular orientation of the silk fibers, thereby significantly enhancing the tensile strength and Young's modulus.

Consistent with the domestication trajectory from wild to domestic silkworms, long-term selective breeding of domestic silkworms has achieved the core goals of easy rearing and high silk yield, accompanied by a substantial increase in fibroin content. However, current silkworm varieties have reached a bottleneck of genetic homogeneity, and further improvements to the silk yield and natural silk fiber properties have stagnated [20,27,44,76]. Therefore, reducing the sericin content of the raw cocoon is the most direct technical approach to improve silk utilization efficiency at the source. The core of cocoon silk processing is to isolate fibroin fibers by utilizing the water-soluble property of sericin. Conventional strategies and technologies for reeling continuous silk filaments for weaving require a sufficiently thick sericin layer [1,19,51]. Interestingly, although the surface sericin content of the *Per*^−/−^ mutant cocoon silk in this study was significantly reduced, the surfaces of the fibroin fibers after degumming under harsh conditions were undamaged, and also smoother than their WT counterparts, which may be associated with changes to the fibrous structure and physical properties of cocoon silk arising from the altered spinning tube morphology and increased spinning rate of the mutant. Notably, GABA-CRZ is not the only neural pathway governing spinning rate. In the spinneret and ASG of spinning-stage *Per*^−/−^ mutants, the transcript level of *Tan*, encoding a hydrolase that regenerates dopamine from N-β-alanyl-dopamine, was significantly upregulated compared with WT silkworms, while that of *Aanat*, encoding the rate-limiting enzyme for dopamine degradation, was downregulated. These observations suggest that dopamine metabolism is also altered in *Per*^−/−^ mutants and may cooperate with the GABA-CRZ pathway to regulate spinning behavior, with potential impacts on silk fiber formation and mechanical performance.

## 5 Conclusion

This study suggests an innovative strategy for silkworm germplasm innovation to break the efficiency bottleneck of cocoon silk production. Loss-of-function mutants of the circadian negative regulator *per* in *B. mori* display significantly improved cocoon silk yield and enhanced mechanical properties, which are associated with altered development of distinct SG regions and spinning behavior. Notably, the proportion of fibroin, the effectively utilizable component in cocoon silk, was significantly increased from 69.92% to 76.33%.

## CRediT authorship contribution statement

**Guang Wang:** Writing – review & editing, Writing – original draft, Visualization, Project administration, Investigation, Funding acquisition, Formal analysis, Data curation, Conceptualization. **Jianfeng Qiu:** Writing – original draft, Validation, Software, Methodology, Investigation, Funding acquisition, Data curation, Conceptualization. **Jianglan Li:** Visualization, Investigation, Formal analysis, Data curation. **Chenchen Wang:** Validation, Methodology, Investigation. **Xinxin Liu:** Validation, Software, Investigation. **Cheng Luo:** Methodology, Investigation. **Ruji Peng:** Software, Investigation. **Taiming Dai:** Methodology, Formal analysis. **Xiaoning Sun:** Visualization, Methodology. **Yanhong Jiang:** Validation, Methodology. **Yanghu Sima:** Supervision, Resources. **Shiqing Xu:** Writing – review & editing, Writing – original draft, Supervision, Resources, Project administration, Funding acquisition, Data curation, Conceptualization.

## Declaration of competing interest

The authors declare that they have no known competing financial interests or personal relationships that could have appeared to influence the work reported in this paper.

## Data Availability

Data will be made available on request.
