# Supplementary material for: Targeting the circadian negative regulator period in Bombyx mori: a strategy for improving silk fibroin yield and mechanical properties
Source: Mater Today Bio. 2026 Sep 4;40:103650. doi: 10.1016/j.mtbio.2026.103650 (PMC13578639; doi:10.1016/j.mtbio.2026.103650)
Supplement: Supplementary file 1 [file mmc1.docx]

Supporting Information for

**Targeting the circadian negative regulator *period* in *Bombyx mori*: a strategy for improving silk fibroin yield and mechanical properties**

Guang Wang ^a,b,1^, Jianfeng Qiu ^a,c,1^, Jianglan Li ^a,b^, Chenchen Wang ^a,b^, Xinxin Liu ^a,b^, Cheng Luo ^a,b^, Ruji Peng ^a,b^, Taiming Dai ^a,b^, Xiaoning Sun ^a,b^, Yanhong Jiang ^a,b^, Yanghu Sima ^a,b^, Shiqing Xu ^a,b,*^

^a^ School of Life Sciences, Suzhou Medical College, Soochow University, Suzhou 215123, China;

^b^ National Engineering Laboratory for Modern Silk, Soochow University, Suzhou 215123, China;

^c^ College of Bee Science, Fujian Agriculture and Forestry University, Fuzhou 350002, China.

^*^ Corresponding author. Email: szsqxu@suda.edu.cn (Xu)

^1^ Guang Wang and Jianfeng Qiu contributed equally to this work.

Corresponding author

Prof. Shiqing Xu

School of Life Sciences, Suzhou Medical College, Soochow University.

Renai Road 199, Dushu Lake Higher Education Town, Suzhou 215123, China.

Tel: +86-512-65880185

Email: szsqxu@suda.edu.cn (Xu)

**1. Materials and methods**

*1.1. Hematoxylin and eosin staining of cocoon silk cross-sections*

The middle layer of the silkworm cocoon was collected on day 7 after spinning onset and fixed in 4% paraformaldehyde (P0099, Beyotime, China) for 24 h. Following a previously described method [1], the fixed samples were sequentially dehydrated in a graded ethanol series, cleared, infiltrated with wax, and embedded in paraffin prior to sectioning. Sections were then dewaxed, rehydrated, stained with hematoxylin and eosin, and mounted with neutral balsam. Images were observed and captured under an optical microscope, and the proportion of fibroin area in the cocoon silk cross-section was analyzed and calculated using ImageJ software.

*1.2. Analysis of cocoon silk yield and relative contents of fibroin and sericin*

On day 7 after spinning onset, cocoon samples were collected to measure single cocoon weight, cocoon shell weight, and pupal weight, with 20 cocoons examined per sex per group. After removal of the outer floss and pupal lining, the cocoon shells were dried at 85°C to constant weight, then boiled in 0.2% sodium carbonate solution for 1 h and rinsed three times to completely remove sericin (*n* = 3 per group per sex). Following drying again at 85°C to constant weight, the weight of fibroin protein was obtained. The weight difference between the sample before degumming and the fibroin was recorded as the weight of sericin removed.

*1.3. Investigation of SG development*

On L5D0, L5D3, and L5D6, body weight and total SG weight were measured, and the MSG and PSG were weighed separately. On L5D3, L5D4, and L5D5, the length of each SG segment was measured. Ten silkworms per sex were used for each time point.

*1.4. Analysis of silk spinning behavior*

Head-swinging frequency within 1 min was recorded at 6 h after spinning onset following the method described [2]. For each larva, the reported value represents the mean of three independent measurements. Silk yield within 24 h after spinning onset was further measured (*n* = 20 per group per sex).

*1.5. Measurement of DNA content in SG*

On L5D3, WT and *Per*^-/-^ larvae of similar body weight were selected. PSG or MSG from three individuals were pooled to form one biological replicate, and three independent replicates were prepared per group. Total DNA was extracted from PSG and MSG samples using DNAiso Reagent (9770A, TaKaRa, China), and DNA concentration was measured using a NanoDrop One/OneC microvolume UV–Vis spectrophotometer (840-317400, Thermo Fisher, USA).

*1.6. RT-PCR and qPCR analyses*

Total RNA was extracted from SG or brain tissue using TRIzol^®^ Reagent (15596018, Thermo Fisher Scientific, USA), and cDNA was synthesized by reverse transcription using the PrimeScript RT reagent Kit (RR037B, TaKaRa, China). qPCR was performed using TB Green Fast qPCR Mix (RR430A, TaKaRa, China) on an ABI StepOnePlus™ real-time PCR system (Applied Biosystems, Foster City, CA, USA), with three replicates per sample. Relative expression levels were calculated using the 2^-ΔΔCt^ method. RT-PCR was conducted using 2× Phanta Flash Master Mix (P520, Vazyme, China). Amplified cDNA products were separated by 1% agarose gel electrophoresis and visualized using a ChemiDoc™ Imaging System (Bio-Rad, USA). The *Bombyx mori Rp49* gene was used as an internal control. Primer sequences used in this studyare listed in Table S3.

*1.7. Transcriptome sequencing and analysis*

On L5D3, PSG were collected from WT and *Per*^-/-^ larvae. Each biological replicate consisted of pooled PSG from five individuals, with three independent replicates per group. Total RNA extraction, transcriptome library construction, and sequencing were performed by Majorbio (Shanghai, China) using the Illumina NovaSeq X Plus platform (Illumina, San Diego, CA, USA). Clean reads from each sample were aligned to the silkworm reference genome (NCBI accession: GCF_030269925.1) using HISAT2 (version 2.2.1). Gene expression levels were quantified as transcripts per million (TPM) using RSEM (version 1.3.3). Differential gene expression analysis between WT and *Per*^-/-^ groups was conducted with DESeq2 (version 1.42.0) using thresholds of |log_2_FC| ≥ 1 and adjusted *P* < 0.05. Differentially expressed genes (DEGs) were subjected to GO functional annotation and KEGG pathway enrichment analysis. GSEA (version 4.3.2) was performed to identify gene sets with coordinated but subtle expression changes.

*1.8. Western blot analysis*

Following a previously described method [3], SG from L5D6 larvae were rapidly frozen in liquid nitrogen, then immersed in pre-cooled 60% ethanol to solidify luminal contents. After removal of the glandular cell layer, luminal contents were rinsed with ultrapure water and dried to obtain luminal silk proteins. Silk proteins were extracted from one SG per larva, pooled from three larvae, and dissolved in 1 mL of 9 mol/L LiSCN solution. After centrifugation, the supernatant was collected, and its concentration was determined using a BCA protein assay kit (P0010, Beyotime, China). Protein samples (50 μg) were separated by 12% SDS-PAGE and transferred onto a PVDF membrane. The membrane was blocked at room temperature for 2 h, then incubated overnight at 4°C with primary antibodies against FIB-H (1:2000), FIB-L (1:2000), P25 (1:2000), or ACTIN (1:1000; sc-47778, Santa Cruz Biotechnology, USA). The membrane was then incubated with horseradish peroxidase (HRP)-conjugated secondary antibodies at 37°C for 2 h. Chemiluminescent signals were detected using Clarity Max Western ECL substrate (1705062, Bio-Rad, USA) with a ChemiDoc™ Touch imaging system (Bio-Rad, USA) and quantified using Image Lab software (Bio-Rad).

*1.9. Gene knockdown*

*Per*^-/-^ female larvae were microinjected 12 h before spinning onset. Each larva in the experimental group was injected with 10 μg of small interfering RNA (siRNA) targeting the glutamic acid decarboxylase gene (*Gad*; Gene ID: 101746611), which was a 1:1 (mass ratio) mixture of siRNA-Gad 1603 and siRNA-Gad 579, designed and synthesized by Sangon Biotech (Shanghai, China). The control group received an equal dose (10 μg) of non-targeting negative control siRNA (siNC). Brain tissues were collected 24 h post-injection, and knockdown efficiency of *Gad* was verified by qPCR analysis.

**2. Results**

*2.1. Normal vitality and development alongside enhanced fibroin production efficiency in the Per^-/-^ mutant*

We previously established a homozygous *per* mutant line *Per*^-/-^ using TALEN technology, with the WT strain Dazao as the control [4]. In the present study, after more than 10 generations of breeding, Sanger sequencing confirmed a 19‑bp insertion in the target gene of the *Per*^-/-^ line, which introduced a premature stop codon downstream of the target site (Fig. S1A, B). RT‑PCR and western blot analyses further confirmed that *per* expression was disrupted at both the transcript and protein levels (Fig. S1C, D). Collectively, these results verified the stability of the mutation site, the homozygous status, and the loss‑of‑function of *per* in this mutant line.

**Figure S1.** Molecular validation of the homozygous *per* knockout mutant line *Per*^-/-^. (A) Schematic structure of the *per* gene. (B) Sequence of the knockout target site. The orange region indicates the TALEN-targeted site. The mutant harbors a 19-bp insertion and a premature stop codon downstream of the target site. Wild-type strain Dazao (WT) was used as a control. (C) RT-PCR analysis of *per* mRNA levels in newly hatched larvae. *B. mori Rp49* served as the internal reference. *n* = 3. (D) Western blot analysis of PER protein. Total protein was extracted from WT and *Per*^-/-^ larvae. α-Tubulin served as the internal reference.

Larval survival of *Per*^-/-^ and WT silkworms exceeded 95% in both the spring and autumn rearing seasons (Fig. S2A). At the 5th instar stage, there was no significant difference in the body weight of male larvae, whereas the body weight of female larvae tended to increase (Fig. S2B, C). Cocoon trait analysis revealed a normal cocooning rate and normal cocoon and pupal morphology of the mature larvae of both silkworm lines (Fig. S2D, E). The cocoon weights of male and female *Per*^-/-^ silkworms were increased by 5.77% and 8.72%, respectively, as compared to the WT, while the pupal weights also tended to increase (Fig. S2F, G). Importantly, the relative content of outer sericin in cocoon silk was significantly lower in the *Per*^-/-^ group than the WT group (Fig. S2H).

**Figure S2.** Traits related to development and cocoon silk production in *Per*^-/-^ silkworms. (A) Larval survival rate. Initial larval numbers were 441 for the *Per*^-/-^ group and 396 for the WT group. (B, C) Body weight of fifth-instar larvae. M, male; F, female. *n* = 40. (D) Morphology of silkworm cocoons and pupae. (E) Cocooning rate. The rate is defined as the percentage of larvae that spun cocoons among all mature larvae. Three independent biological replicates were performed per group, with 100 individuals per replicate. (F, G) Cocoon weight and pupal weight. *n* = 20. (H) Relative sericin content of cocoon silk. *n* = 3. (I) Morphology of cocoons at 0, 12, and 24 h after spinning onset. (J, K) Total silk yield and the proportion of silk produced within 24 h after spinning onset relative to total yield. *n* = 20. Data are presented as mean ± SD. ns, *P* > 0.05; ^*^*P* ≤ 0.05, ^**^*P* ≤ 0.01, ^***^*P* ≤ 0.001.

TEM of silk fiber cross-sections revealed a greater abundance of inner fibroin in silk in the *Per*^-/-^ group than the WT group (Fig. S3A). Hematoxylin-eosin staining further showed that the fibroin area in silk was significantly greater in the *Per*^-/-^ group than the WT group (Fig. S3B, C). These observations further confirmed enhanced fibroin production efficiency in the *Per*^-/-^ mutant.

To explore the potential mechanism responsible for the enhanced mechanical properties of *Per*^-/-^ silk fibers (Fig. 2C), the microstructure of the spinneret during the spinning period was examined. Observations revealed that the common tube and pressing zone of *Per*^-/-^ silkworms were relatively more elongated and narrower (Fig. S3D), indicating that these structural changes in the mutant may modulate fibroin assembly by altering the spinning process, such as the silk dope flow rate and shear force. Mechanical analysis further revealed that the improved toughness of *Per*^-/-^ raw silk threads which was associated with significant increases in tensile strength and Young's modulus (Fig. 2D, F), whereas the elongation at breakage remained unchanged (Fig. S3E). Further nanostructural analysis showed that the one-dimensional SAXS intensity distribution of *Per*^-/-^ silk fibers in the q range of 0.008–0.06 Å⁻¹ differed significantly from that of the WT group (Fig. S3F), indicating altered nanostructure at this characteristic scale.

**Figure S3.** Cocoon silk structure and composition in *Per*^-/-^ silkworms. (A) TEM images of silk fiber cross-sections. WT-C and *Per*^-/-^-C represent cross-sections of WT and *Per*^-/-^ silk fibers, respectively. SF, silk fibroin; SS, silk sericin. (B, C) Hematoxylin and eosin staining of silk fiber cross‑sections and SF layer area ratio. *n* = 20. (D) Anatomical diagram of the spinneret in a mature larva. ASG, anterior SG. Spinnerets were dissected 6 h after spinning onset. (E) Elongation at break of raw silk threads. *n* = 30. (F) One-dimensional SAXS scattering profiles of silk protein samples (log–log scale). Curves show scattering intensity of WT and *Per*^-/-^ samples in the q range of 0–0.45 Å⁻¹. The dashed box indicates the region corresponding to key differences highlighted in Fig. 2N. Data are presented as mean ± SD. ns, *P* > 0.05; ^***^*P* ≤ 0.001.

*2.2. Enhanced PSG development and fibroin synthesis and secretion in the Per^-/-^ mutant*

During the 5th instar stage, a critical time for silk protein synthesis, morphological observation of the SG showed no significant differences between the two groups, as both exhibited typical and well-developed structures (Fig. S4A). In male larvae, except for the significantly higher SG weight and PSG/body weight ratio observed in the *Per*^-/-^ group at L5D0, no significant difference in SG weight, SG/body weight ratio, or PSG/body weight ratio was detected between the two groups at the early and middle stages (L5D0 and L5D3). However, at the late stage (L5D6), these three parameters were significantly higher in the *Per*^-/-^ group than the WT group (Fig. S4B–D).

Further analysis of relative MSG and PSG weights, expressed as percentages of the total SG weight, revealed that throughout the sampling period, the PSG proportion was consistently significantly higher in the *Per*^-/-^ group than the WT group (Fig. S4E), whereas the MSG proportion was consistently lower (Fig. S4F). This trend was consistent in female larvae (Fig. 3B–F). In addition, the length of the posterior region of the MSG was significantly shorter in the *Per*^-/-^ group than the WT group during the middle and late 5th instar stages (Fig. S4G). These results further confirmed enhanced PSG development in the *Per*^-/-^ mutant.

**Figure S4.** Enhanced development of the PSG in *Per*^-/-^ silkworms. (A) Local morphology of SGs in 5th instar larvae. (B–F) Developmental indices of SGs in 5th instar male larvae. (B) SG weight. (C) SG-to-body weight ratio. (D) PSG-to-body weight ratio. (E) PSG-to-SG weight ratio. (F) MSG-to-SG weight ratio. (G) Posterior MSG (pMSG) length. *n* = 10. Data are presented as mean ± SD. ns, *P* > 0.05; ^*^*P* ≤ 0.05, ^**^*P* ≤ 0.01, ^***^*P* ≤ 0.001.

Based on RNA-seq analysis of the PSG from larvae at L5D3, GSEA revealed that gene sets associated with "RNA-DNA hybrid ribonuclease activity" and "endoribonuclease activity, producing 5′-phosphomonoesters" were upregulated in the *Per*^-/-^ group (Fig. S5A–C). EdU staining of PSG tissue also revealed significantly higher fluorescence intensity in the *Per*^-/-^ group than the WT group (Fig. S5D). In addition, KEGG enrichment analysis revealed that DEGs upregulated in the *Per*^-/-^ group were significantly enriched in the "Protein processing in endoplasmic reticulum" pathway (Fig. S5E). These results confirmed enhanced DNA replication and protein synthesis in the PSG of the *Per*^-/-^ mutant.

**Figure S5.** Upregulated expression of fibroin genes in *Per*^-/-^ silkworms. (A) L5D3 larvae. (B, C) GSEA showing significantly upregulated gene sets in *Per*^-/-^ compared with WT. (D) EdU fluorescence intensity density in the PSG. Density was calculated as total integrated intensity of EdU-positive signals divided by total tissue area per field of view. (E) KEGG enrichment analysis of significantly upregulated genes in *Per*^-/-^ compared with WT. (F, G) Silk protein gene transcript levels in the SG analyzed by qPCR during the fifth instar. (F) Fibroin genes *Fib-H*, *Fib-L*, and *P25*. (G) Sericin genes *Ser1*, *Ser2*, and *Ser3*. (H) mRNA levels of key transcriptional regulators of silk protein genes in the MSG and PSG during the fifth instar analyzed by qPCR. *Rp49* served as the internal reference. *n* = 3. Data are presented as mean ± SD. ns, *P* > 0.05; ^*^*P* ≤ 0.05, ^**^*P* ≤ 0.01, ^***^*P* ≤ 0.001.

qPCR analysis of silk protein gene transcript levels in the SG during the 5th instar stage revealed that the expression of fibroin genes (*Fib-H*, *Fib-L*, and *P25*) was overall significantly upregulated in the *Per*^-/-^ group as compared to the WT group, except for *Fib-H* at L5D3 (no significant difference) (Fig. S5F). In contrast, expression of sericin genes (*Ser1*, *Ser2*, and *Ser3*) was significantly downregulated at L5D0 and L5D3 (except for *Ser2* at L5D3), and returned to WT levels at L5D6 (Fig. S5G). These results showed that relatively higher fibroin and lower sericin transcript proportions within the total SG of the *Per*^-/-^ mutant.

Further analysis of key regulators of silk protein expression in different functional regions of the SG revealed that transcript levels of the positive sericin regulators *Sgf1* and *Sgf3* were significantly lower in the MSG of the *Per*^-/-^ group than the WT group during the early and middle 5th instar stages, consistent with the expression pattern of sericin genes, and exceeded or returned to WT levels at L5D6 (Fig. S5H). In the PSG, although the transcript levels of the positive fibroin regulators *Sage*, *Dimm*, and *Sgf2* were lower in the *Per*^-/-^ group than the WT group at L5D0, the levels increased continuously thereafter and were all significantly higher at L5D6. Expression of the negative regulator *Fmbp1* was significantly lower in the *Per*^-/-^ group than the WT group at L5D0 and gradually decreased thereafter. Although slightly higher than WT levels at L5D6, the inhibitory effect of *Fmbp1* on fibroin was considerably limited at this stage (Fig. S5H). These results were consistent with enhanced fibroin gene transcription in the *Per*^-/-^ mutant during the middle and late 5th instar stages, a period of rapid silk protein synthesis.

To identify molecular links between circadian signaling and fibroin synthesis, we found that *Jhamt-like* (juvenile hormone acid methyltransferase-like gene), which is directly regulated by the circadian clock, was significantly upregulated in the PSG of the *Per*^-/-^ group (Fig. S6A). This was accompanied by higher transcript levels of the JH receptor, *Met2*, and its downstream target, *Kr‑h1*, relative to the WT group during the middle 5th instar stage (Fig. S6B). Meanwhile, the transcript levels of endoplasmic reticulum protein processing‑related genes *Ugt* and *Man1* were also significantly elevated in SG cells during the middle and late 5th instar stages (Fig. S6C, D). These results revealed JH signaling activation alongside enhanced PSG development and fibroin synthesis in the *Per*^-/-^ mutant.

In addition, we also examined key genes involved in dopamine metabolism in the spinneret and ASG at the spinning stage. Compared with WT, *Tan*, encoding a hydrolase that regenerates dopamine, was significantly upregulated, whereas *Aanat*, encoding the rate‑limiting enzyme for dopamine degradation, was markedly downregulated in *Per*^-/-^ mutants (Fig. S6I–L). No obvious change was detected for the dopamine‑synthetic gene *Ddc*. These data suggest that dopamine metabolism may also be altered in the *Per*^-/-^ mutant.

**Figure S6.** Promoted synthesis and accelerated secretion of silk proteins in *Per*^-/-^ silkworms. (A) RNA-seq analysis showing the differential expression of the *Jhamt-like* gene. (B) Transcript levels of the JH receptor *Met2* and downstream target gene *Kr-h1* on L5D3 analyzed by qPCR. (C, D) Transcript levels of endoplasmic reticulum pathway-related genes *Ugt* and *Man1* in the SG on L5D3 and L5D6 analyzed by qPCR. *Rp49* served as the internal reference gene. For A–D, *n* = 3. (E, F) PSG-to-body weight ratio during the spinning stage. (G, H) MSG-to-body weight ratio during the spinning stage. For E–H, *n* = 10. W0 and W24 denote 0 and 24 h after spinning onset, respectively. (I) Schematic representation of the dopamine metabolic pathway. DOPA, 3,4-dihydroxyphenylalanine; DA, dopamine; NADA, N‑acetyl‑dopamine; NBAD, N-β-alanyl‑dopamine; DDC, dopa decarboxylase; AANAT, arylalkylamine N‑acetyltransferase; EBONY, NBAD synthase; TAN, NBAD hydrolase. (K–L) Transcript levels of key enzyme-encoding genes in the dopamine metabolic pathway in spinning-stage silkworms analyzed by qPCR. For J–L, *n* = 3. Data are presented as mean ± SD. ns, *P* > 0.05; **P* ≤ 0.05, ***P* ≤ 0.01, ****P* ≤ 0.001.

*2.3. Accelerated silk spinning rate associated with GABA-CRZ signaling in the Per^-/-^ mutant*

At the spinning stage, investigation of SG weight revealed that (Fig. S6E–H), regardless of sex, there was no significant difference in the MSG/body weight ratio at 0 h after spinning onset between the *Per*^-/-^ and WT groups, whereas the PSG/body weight ratio was significantly higher in the *Per*^-/-^ group, further confirming enhanced PSG development and greater fibroin accumulation in *Per*^-/-^ larvae. At 24 h after spinning onset, the MSG/body weight ratio and PSG/body weight ratio were significantly lower in the *Per*^-/-^ group than the WT group, consistent with accelerated silk fluid discharge from the SG in the *Per*^-/-^ mutant.

Changes to the GABA content of the brain and hemolymph at 0–24 h after spinning onset were determined with an enzyme-linked immunosorbent assay. GABA levels gradually increased over time in the WT brain, but remained consistently high in the *Per*^-/-^ brain (Fig. 5F). In contrast, the GABA concentration in *Per*^-/-^ hemolymph was persistently low, at only 70.89% and 79.06% of WT levels at 0 and 24 h, respectively (Fig. S7A). Further investigation of key gene expression in the GABAergic neural signaling pathway revealed that, at 12 and 24 h after spinning onset, the transcript levels of *Vgat* (vesicular GABA transporter) and the GABA receptors *Rdl1* and *Rdl2* in the brain were significantly higher in the *Per*^-/-^ group than the WT group (Fig. S7B–D). These results suggested enhanced brain GABA synthesis and diminished release into the hemolymph in the *Per*^-/-^ mutant.

**Figure S7.** Effects of GABA-CRZ signaling on cocoon silk production in *Per*^-/-^ silkworms. (A) GABA content in the silkworm hemolymph during the spinning stage. W0, W12, and W24 denote 0, 12, and 24 h after spinning onset, respectively. (B–D) Transcription levels of GABA signaling-related genes *Vgat*, *Rdl1*, and *Rdl2* in the brain during the spinning stage analyzed by qPCR. *Rp49* served as the internal reference gene. *n* = 3. (E–I) Effects of *Gad* knockdown or CRZ supplementation on cocoon silk morphology in *Per*^-/-^ mutants. (E) SEM images of cocoon silk. (F) Relative sericin content of cocoon silk. *n* = 5. (G) Total silk yield. *n* = 20. (H) Proportion of silk produced within the first 24 h after spinning onset relative to total yield. *n* = 20. (I) Elongation at break of raw silk threads. *n* = 30. *Per*^-/-^ female larvae were injected with 10 µg siGad or 1 nmol CRZ at 12 h before spinning onset (after feeding cessation). Control larvae received an equal volume of siNC or ultrapure water. Data are presented as mean ± SD. ns, *P* > 0.05; ^*^*P* ≤ 0.05, ^**^*P* ≤ 0.01, ^***^*P* ≤ 0.001.

To explore the role of GABA-CRZ signaling in regulating the spinning rate and cocoon traits of *Per*^-/-^ silkworms, siGad or CRZ was injected into *Per*^-/-^ larvae at 12 h before spinning onset, whereas the control groups were injected with siNC or ultrapure water, respectively. The results showed that knockdown of *Gad* or exogenous supplementation of CRZ tightened adhesion between silk fibers, reduced the pore size, and increased the sericin content of cocoon silk (Fig. S7E, F). Analysis of the spinning rate revealed that although knockdown of *Gad* or exogenous CRZ supplementation had no effect on the total silk yield, the proportion of silk produced within the first 24 h of spinning was significantly reduced (Fig. S7G, H). In addition, breaking elongation of raw silk threads was increased, but not significantly (Fig. S7I).

*2.4. Analysis of sericin content variation in cocoon silk across silkworm varieties*

The domesticated silkworm *B. mori* originated from the wild silkworm *B. mandarina*. Long-term domestication and breeding selection have increased silk yield, and also altered cocoon silk composition and properties [5,6]. Transcriptome analysis of the SGs of domesticated and wild silkworm larvae revealed that upregulated DEGs in domesticated silkworms are mainly involved in efficient synthesis and secretion of silk proteins, whereas those upregulated in wild silkworms are primarily associated with pathogen tolerance and environmental adaptation [7]. These findings indicate that during domestication, physiological resource allocation in domesticated silkworms shifted from immune defense and environmental adaptation, as observed in wild silkworms, toward the efficient synthesis of silk proteins.

Analysis of cocoon silk from domesticated and wild silkworms, as well as varieties with different voltinism, revealed that wild silkworm cocoons had a much higher sericin content than domesticated silkworm cocoons, and that among domesticated varieties, multivoltine strains generally exhibited higher sericin content than bivoltine strains, which in turn had higher levels than univoltine strains. In F_1_ hybrids derived from reciprocal crosses between the practical varieties Jingsong and Haoyue, silk yield increased, while cocoon silk sericin content decreased as compared to the parental strains. This trend is consistent with the reduction in sericin content observed during domestication from wild to domesticated silkworms. In addition, no significant difference in cocoon silk sericin content was detected between female and male cocoons (Table S1). These results suggested that higher sericin content favored ecological adaptation of silkworms in natural environments.

Further analysis of 33 bivoltine silkworm germplasm resources revealed a narrow variation in cocoon silk sericin content among varieties, indicating that long-term domestication and selective breeding have led to a bottleneck of genetic homogenization, with fibroin and sericin compositions becoming stable and highly similar in current silkworm strains (Table S2). Therefore, elucidating the mechanisms and establishing strategies to enhance fibroin content, while maintaining stable silk yield, is critical to overcoming the breeding bottleneck and meeting industrial demands for fibroin.

**Table S1.** Sericin content in the cocoon shell of silkworm genetic resources by voltinism.

| Reference | Year | Authors | Silkworm materials | Category | Sericin content (%) |
| --- | --- | --- | --- | --- | --- |
| 5 | 2019 | Dai et al. | *Bombyx mandarina* | Wild silkworm | 33.34 |
|  |  |  | *Bombyx mori* (Xiafang) | Domestic silkworm | 27.02 |
| 8 | 2012 | Liu et al. | Dazao | Bivoltine | 34.77 |
|  |  |  | Flossy | Bivoltine | 29.61 |
|  |  |  | Xiafang | Bivoltine | 27.18 |
| 9 | 2009 | Qu et al. | Guican 1 | Multivoltine | 31.17 |
|  |  |  | Liangguang 2 | Multivoltine | 29.95 |
|  |  |  | Jingsong×Haoyue | Bivoltine | 27.31 |
|  |  |  | Qiuju×Minghu | Bivoltine | 26.65 |
| 10 | 1998 | Li et al. | C_11_ (♀) | Parental Line | 28.69 |
|  |  |  | Jingsong (♀) | Parental Line | 28.31 |
|  |  |  | Haoyue (♀) | Parental Line | 26.85 |
|  |  |  | Haoyue×Jingsong (♀) | F1 Hybrid | 25.91 |
|  |  |  | Jingsong×Haoyue (♀) | F1 Hybrid | 25.54 |
|  |  |  | J_1_×Haoyue (♀) | F1 Hybrid | 23.22 |
|  |  |  | C_11_ (♂) | Parental Line | 28.66 |
|  |  |  | Jingsong (♂) | Parental Line | 28.24 |
|  |  |  | Haoyue (♂) | Parental Line | 26.86 |
|  |  |  | Haoyue×Jingsong (♂) | F1 Hybrid | 25.91 |
|  |  |  | Jingsong×Haoyue (♂) | F1 Hybrid | 25.52 |
|  |  |  | J_1_×Haoyue (♂) | F1 Hybrid | 23.23 |
| 11 | 1996 | Lin et al. | Chinese Bivoltine (94 strains) | Bivoltine | 26.9 |
|  |  |  | Japanese Bivoltine (74 strains) | Bivoltine | 25.8 |
|  |  |  | European Univoltine (39 strains) | Univoltine | 24.4 |
|  |  |  | Chinese Univoltine (4 strains) | Univoltine | 23.9 |
|  |  |  | Japanese Univoltine (11 strains) | Univoltine | 22.3 |

**Table S2.** Sericin content in the cocoon shell of different silkworm genetic resources.

| Silkworm strain | Silkworm seasonal type | Hybrid system | Sericin content (%) |
| --- | --- | --- | --- |
| 9804×9803 | Spring silkworm | F1 hybrid | 31.62 |
| Zabai×Jingsong | Spring silkworm | F1 hybrid | 31.44 |
| Haoyue×Jingsong | Spring silkworm | F1 hybrid | 31.25 |
| 9805×9806 | Spring silkworm | F1 hybrid | 30.35 |
| 9803×9804 | Spring silkworm | F1 hybrid | 30.23 |
| Haoyue×I0529 | Spring silkworm | F1 hybrid | 29.36 |
| Fa408.8712×Jingsong | Spring silkworm | Three-way cross | 30.26 |
| 9442-Bai×I0533 | Summer silkworm | F1 hybrid | 34.17 |
| 0804-Hua×I-1005 | Summer silkworm | F1 hybrid | 32.60 |
| 107×7532 | Summer silkworm | F1 hybrid | 32.58 |
| 9442-Xian×75-Jingxian | Summer silkworm | F1 hybrid | 31.96 |
| 0822×873 | Summer silkworm | F1 hybrid | 31.78 |
| 7532×75-Xin | Summer silkworm | F1 hybrid | 31.43 |
| 0802H×0821-Kang | Summer silkworm | F1 hybrid | 31.42 |
| 0804-Hua×7533-Xian | Summer silkworm | F1 hybrid | 30.56 |
| 75Xin×7532 | Summer silkworm | F1 hybrid | 30.30 |
| 0804×C7 | Summer silkworm | F1 hybrid | 30.18 |
| 0805×0804-Hua | Summer silkworm | F1 hybrid | 30.16 |
| 0802H×I-1005 | Summer silkworm | F1 hybrid | 30.10 |
| I-1001×0704 | Summer silkworm | F1 hybrid | 29.78 |
| 0804-Hua×0805 | Summer silkworm | F1 hybrid | 29.67 |
| 9442-Xian×I-1013 | Summer silkworm | F1 hybrid | 28.57 |
| 8411.I-1001×9442-Xian | Summer silkworm | Three-way cross | 33.67 |
| I-1001.C7×0704 | Summer silkworm | Three-way cross | 32.54 |
| I-1001.C7×0804-Hua | Summer silkworm | Three-way cross | 32.14 |
| I-1001.C7×9432-Xian | Summer silkworm | Three-way cross | 31.79 |
| 0804-Bai×8411. I-1001 | Summer silkworm | Three-way cross | 30.56 |
| 9442-Xian×8411. I-1001 | Summer silkworm | Three-way cross | 29.38 |
| 8418.9446×9405.8725 | Summer silkworm | Double cross | 31.43 |
| 0704×9431 | Autumn silkworm | F1 hybrid | 31.91 |
| 9431×9442-Xian | Autumn silkworm | F1 hybrid | 29.17 |
| 9442×0713 | Autumn silkworm | F1 hybrid | 29.02 |
| 9442-Xian×9431 | Autumn silkworm | F1 hybrid | 28.90 |

**Table S3.** Primers used in this study.

| **Using** | **Genes** | **Primers** | **Sequences (5′ to 3′)** |
| --- | --- | --- | --- |
| RT-PCR | *Period* | Per-F | GAAACGGAAACTGTATCGC |
|  |  | Per-R | GAGGCAACAGAAGTAGTCA |
|  | *Fibroin-H* | Fib-H-F | GGGAGTGATGTCACTGTCCA |
|  |  | Fib-H-R | ACTCCTTGTCCGTACCCA |
|  | *Fibroin-L* | Fib-L-F | GTCGATGGGACTACGT |
|  |  | Fib-L-R | ATTGGTGGAACACTTGA |
|  | *P25* | P25-F | CTGCTACTTGGACGATT |
|  |  | P25-R | CAGCCAGTTCCTCTTT |
|  | *Sericin1* | Ser1-F | TGCAAGCTCAAACAGCGTAG |
|  |  | Ser1-R | CGGACGTGGTGGATTCTTCT |
|  | *Sericin2* | Ser2-F | GCGTGGCTGTGGTCAA |
|  |  | Ser2-R | GCCGTCGCTGTCGTTA |
|  | *Sericin3* | Ser3-F | ATGTCTCGTCGGTGGA |
|  |  | Ser3-R | CTGCTGTCGTCGGAAT |
|  | *Rp49* | Rp49-F | ACTCTGATGCTGAGCTGCTG |
|  |  | Rp49-R | GACCTGTTTACAGGCCGACA |
| qPCR | *Clock* | Clk-F | TGAACTCACATCCTGCTACT |
|  |  | Clk-R | CTTTCTTGCTTGGCGTTT |
|  | *Cycle* | Cyc-F | AAACGGAAACCATCGTCCTA |
|  |  | Cyc-R | TTTGTTTCTTGTCGGGAGTG |
|  | *Timeless* | Tim-F | CTCTGCTCGGTCTTGTCATT |
|  |  | Tim-R | TGCACGGCTTGAGACCATTA |
|  | *Cryptochrome 2* | Cry2-F | TTGTCCGTGAAGAGAGAGCG |
|  |  | Cry2-R | AGAGAGAAAAGCCTGGGTGG |
|  | *Gat* | Gat-F | TCTTCAATCTCGTGCAGTGG |
|  |  | Gat-R | GTGACACGCCACAGGTACAC |
|  | *Gad* | Gad-F | TACCAAGTCAAAACCGGGCA |
|  |  | Gad-R | ATCAAGATGAAGACCGGGGC |
|  | *Grd* | Grd-F | TTATCCTTGGCAGCTACGCC |
|  |  | Grd-R | CGGTGTGCCTTTTGAGGTTG |
|  | *Rdl1* | Rdl1-F | TCCAGAATGCCCTCCAG |
|  |  | Rdl1-R | AAAACGGACTTCAGATGGTCT |
|  | *Rdl2* | Rdl2-F | TACCACCTAGCCGATCTTCG |
|  |  | Rdl2-R | TTGTCCTCCTGCTTCTTCGT |
|  | *Vgat* | Vgat-F | GGATGGAATTGCTCCAAGAA |
|  |  | Vgat-R | TTTTAGCTCCTGGCTTTCCA |
|  | *CrzR* | CrzR-F | TTCTGAATCCAGACAAAAACCAA |
|  |  | CrzR-R | ATGATGTGTTGTTATCACCACGA |
|  | *Fibroin-H* | Fib-H-F | ACAAGGTGCAGGAAGTGC |
|  |  | Fib-H-R | AGCAATTCACACAAGGCAGT |
|  | *Fibroin-L* | Fib-L-F | CCGGAGGTGGAAGAATCTAT |
|  |  | Fib-L-R | GGTTATGTAGGCAGCGATGT |
|  | *P25* | P25-F | CCCTGCTACTTGGACGATT |
|  |  | P25-R | GATTATGGTCGACGTAGGTG |
|  | *Sericin1* | Ser1-F | CCTCTGTAAGCAGTAAGGATAG |
|  |  | Ser1-R | TTGTTTGAGTAAGCAACGGTG |
|  | *Sericin2* | Ser2-F | CCAGGAGGATAACGACAGC |
|  |  | Ser2-R | TGGAGAACTTGTCGTGGGT |
|  | *Sericin3* | Ser3-F | CTGCGGGTACAGGTATG |
|  |  | Ser3-R | ATCGGAGTCCTCGTCAT |
|  | *SGF1* | SGF1-F | AGCACCCGTTCAGCATCA |
|  |  | SGF1-R | GCGGCGACTGGTAGTAGTTAT |
|  | *SGF3* | SGF3-F | CGTCCGCTCAAGAAATCA |
|  |  | SGF3-R | GTTCGGTGGCGTCATCC |
|  | *Sage* | Sage-F | AGCAATCACGAAGGTCCGC |
|  |  | Sage-R | CGTATCGTGGTTGGAGTCGT |
|  | *Dimm* | Dimm-F | CGTGGAACCCGCATTTGTA |
|  |  | Dimm-R | AACCTCGGCAATCCAGTCG |
|  | *SGF2* | SGF2-F | ATGAAGACGGAGCACCGCA |
|  |  | SGF2-R | TTGTGATAGCCCTCCGAGTCAC |
|  | *Fmbp1* | Fmbp1-F | GAAGGAAAGGCTCAGGAC |
|  |  | Fmbp1-R | CGGACATACGCTTCAGT |
|  | *Met2* | Met2-F | ACGGCCATTAAATCCTTG |
|  |  | Met2-R | GGTAAACCCTCACGACAC |
|  | *Krh1* | Krh1-F | ACCCATACTGGCGAGCGACCAT |
|  |  | Krh1-R | CCTCTCCTTTGTGTGAATACGACGG |
|  | *Ugt* | Ugt-F | TTGGCTCAACGATCTGGAGT |
|  |  | Ugt-R | ACGCGTCTCCTTTTGTTGAC |
|  | *Manl* | Manl-F | TCACCCCTTCCAGATGACAC |
|  |  | Manl-R | AAAGCTTGGCCTTCTTGAGC |
|  | *Rp49* | Rp49-F | GCATCAATCGGATCGCTATG |
|  |  | Rp49-R | GGACCTTACGGAATCCATTTG |

**Video S1.** Head-swinging frequency is higher in *in Per*^-/-^ silkworms during the spinning stage. The movie condenses 1 minute of real-time behavior into 30 seconds at 2× speed. WT, wild type; *Per*^-/-^, *period* knockout mutant.

**Video S2.** *Gad* knockdown reduces head-swinging frequency in *Per*^-/-^ silkworms during the spinning stage. The movie condenses 1 minute of real-time behavior into 30 seconds at 2× speed. *Per*^-/-^+siNC, control *Per*^-/-^ mutant; *Per*^-/-^+siGad, *Gad* knockdown in *Per*^-/-^ mutant.

**Video S3.** CRZ supplementation reduces head-swinging frequency in *Per*^-/-^ silkworms during the spinning stage. The movie condenses 1 minute of real-time behavior into 30 seconds at 2× speed. *Per*^-/-^+H₂O, control *Per*^-/-^ mutant; *Per*^-/-^+CRZ, CRZ supplementation in *Per*^-/-^ mutant.

References

[1] Y. Wang, Y. Wang, Q. Li, R. Peng, X. Liang, X. Chen, G. Wang, Y. Sima, S. Xu, Mechanism of differential efficiency in expression of sericin 3 and silk fibroin heavy chain-like protein in the posterior silk gland of the domestic silkworm (*Bombyx mori*), J. Insect Physiol. 168 (2026) 104926. doi: 10.1016/j.jinsphys.2025.104926.
[2] Y. Tanaka, Y. Hua, L. Roller, S. Tanaka, Corazonin reduces the spinning rate in the silkworm, *Bombyx mori*, J. Insect Physiol. 48 (7) (2002) 707–714. doi: 10.1016/s0022-1910(02)00094-x.
[3] Z. Dong, P. Zhao, Y. Zhang, Q. Song, X. Zhang, P. Guo, D. Wang, Q. Xia, Analysis of proteome dynamics inside the silk gland lumen of *Bombyx mori*, Sci. Rep. 6 (2016) 21158. doi: 10.1038/srep21158.
[4] W. Z. Cui, J. F. Qiu, T. M. Dai, Z. Chen, J. L. Li, K. Liu, Y. J. Wang, Y. H. Sima, S. Q. Xu, Circadian clock gene *Period* contributes to diapause via GABAeric-diapause hormone pathway in *Bombyx mori*, Biology (Basel) 10 (9) (2021) 842. doi: 10.3390/biology10090842.
[5] Z. J. Dai, W. Sun, Z. Zhang, Comparative analysis of iTRAQ-based proteomes for cocoons between the domestic silkworm (*Bombyx mori*) and wild silkworm (*Bombyx mandarina*), J. Proteomics 192 (2019) 366–373. doi: 10.1016/j.jprot.2018.09.017.
[6] J. Y. Li, F. Cai, X. G. Ye, J. S. Liang, J. K. Li, M. Y. Wu, D. Zhao, Z. D. Jiang, Z. Y. You, B. X. Zhong, Comparative proteomic analysis of posterior silk glands of wild and domesticated silkworms reveals functional evolution during domestication, J. Proteome Res. 16 (7) (2017) 2495–2507. doi: 10.1021/acs.jproteome.7b00077.
[7] S. M. Fang, B. L. Hu, Q. Z. Zhou, Q. Y. Yu, Z. Zhang, Comparative analysis of the silk gland transcriptomes between the domestic and wild silkworms, BMC Genomics 16 (1) (2015) 60. doi: 10.1186/s12864-015-1287-9.
[8] C. Liu, X. N. Zhu, R. F. Liu, J. B. Ren, H. Shi, L. J. Xun, Q. Y. Xia, Investigation of mutant character flossy in the silkworm, *Bombyx mori*, Newsl. Sericult. Sci. 32 (1) (2012) 1–7.
[9] D. C. Qu, J. Z. He, J. Li, W. J. Lu, J. L. Huang, Determination and analysis of quality and capability index of silkworm cocoon filament in Guangxi, Genom. Appl. Biol. 28 (5) (2009) 921–928. doi: 10.3969/gab.028.000921.
[10] K. R. Li, S. Y. Qi, S. Yao, Z. Q. Zhou, Study on sericin cocoon of different variety of silkworm, *Bombyx mori*, J. Anhui Agric. Univ. 25 (1) (1998) 60–62. doi: 10.13610/j.cnki.1672-352x.1998.01.014.
[11] C. Q. Lin, K. P. Chen, D. X. Wu, P. Wang, Q. Yao, Investigation and correlation analysis of the sericin ratio in the cocoon layer of silkworm (*Bombyx mori*) varietal resources, Acta Sericol. Sin. 2 (1996) 118–120. doi:10.13441/j.cnki.cykx.1996.02.012.
